# The role of USP7-YY1 interaction in promoting colorectal cancer growth and metastasis

**DOI:** 10.1038/s41419-024-06740-4

**Published:** 2024-05-20

**Authors:** Zhi-Ying Shao, Wen-Dong Yang, Hui Qiu, Zhi-Hong He, Meng-Ru Lu, Qi Shen, Jin Ding, Jun-Nian Zheng, Jin Bai

**Affiliations:** 1 Cancer Institute, Xuzhou Medical University, Xuzhou, Jiangsu China; 2 Department of Clinical Trial, Zhejiang Cancer Hospital, Hangzhou Institute of Medicine (HIM), Chinese Academy of Sciences, Hangzhou, Zhejiang China; 3 Center of Clinical Oncology, the Affiliated Hospital of Xuzhou Medical University, Xuzhou, Jiangsu China; 4 Department of Obstetrics and Gynecology, Renji Hospital, Shanghai Jiao Tong University School of Medicine, Shanghai, China; 5 Shanghai Key Laboratory of Gynecologic Oncology, Renji Hospital, School of Medicine, Shanghai Jiaotong University, Shanghai, China; 6 Department of Gastroenterology, Affiliated Jinhua Hospital, Zhejiang University School of Medicine, Jinhua, Zhejiang China; 7 Jiangsu Center for the Collaboration and Innovation of Cancer Biotherapy, Cancer Institute, Xuzhou Medical University, Xuzhou, Jiangsu China

**Keywords:** Cancer, Preclinical research

## Abstract

Colorectal cancer (CRC) remains a significant global health issue with high incidence and mortality. Yin Yang 1 (YY1) is a powerful transcription factor that acts dual roles in gene activation and repression. High expression level of YY1 has been reported in CRC, indicating the existence of stable factors of YY1 in CRC cells. We aimed to identify the key molecules and underlying mechanisms responsible for stabilizing YY1 expression in CRC. Mass spectrometry analysis was utilized to identify USP7 as a potential molecule that interacted with YY1. Mechanically, USP7 stabilizes YY1 expression at the protein level by interfering its K63 linkage ubiquitination. YY1 exerts its oncogenic function through transcriptionally activating TRIAP1 but suppressing LC3B. In addition, at the pathological level, there is a positive correlation between the expression of YY1 and the budding of CRC. This study has revealed the intricate interplay between YY1 and USP7 in CRC, suggesting that they could serve as novel therapeutic targets or predictive biomarkers for CRC patients.

## Introduction

Ranking third in incidence and second in mortality, colorectal cancer (CRC) poses a major global health concern [1]. Due to its tendency to invade and metastasize, over 50% of patients endure metastatic diseases upon initial consultations, resulting in unfavorable survival rates [1, 2]. Hence, unraveling the molecular mechanisms that drive CRC progression is crucial for advancing the development of innovative therapeutic strategies.

Yin Yang 1 (YY1), also known as nuclear factor-E1 (NF-E1), is a transcription factor that contains four conserved C2H2 zinc fingers. Previous investigations have uncovered the dual function of YY1 in gene activation and repression [3–5]. Acting as a multifunctional protein, YY1 participates in various biological and physiological processes, including but not limited to lineage specification, embryonic development, cell proliferation, and tumorigenesis [6, 7]. An exceptionally high level of YY1 expression has been documented in CRC, indicating the presence of stable factors of YY1 in CRC cells [8]. The discovery of these stable factors could offer a promising strategy to reduce YY1 levels within CRC cells.

Ubiquitin-specific protease 7, also known as USP7 or HAUSP, belongs to a group of approximately 100 deubiquitinating enzymes responsible for eliminating ubiquitin (Ub) from specific target proteins and safeguarding them from undergoing degradation [9, 10]. USP7 is a well-studied member of the USP family and has been found to play a role in regulating signaling pathways, inflammation, and stem cell fate. It targets various substrates, including MDM2 (p53 regulator mouse double minute 2 homolog), FOXO4 (Forkhead box O4), REST (RE1-silencing transcription factor), and Aurora A kinase [11–13]. Research on USP7 inhibitors has spanned more than a decade, resulting in the development of over 25 inhibitors with different levels of specificity and potency [9, 11]. In CRC, only a limited number of studies addressed the role of USP7 in enhancing the stem cell property of cancer cells by stabilizing JUND (JunD proto-oncogene) and promoting tumor development by mediating the stability of β-catenin [14–16].

For the first time, our study reveals a fascinating discovery regarding the unique association between YY1 and USP7 in CRC. This interaction may potentially exert a crucial influence on the malignant advancement of CRC by regulating the stability of YY1. Interrupting the combination of YY1 and USP7 may antagonize the proliferation and metastasis of CRC to a certain extent, and provide new ideas and targets for the treatment of CRC.

## Materials and methods

### Cell culture and treatment

CRC cell lines (HCT116, LOVO, and DLD1) and human embryonic kidney cell line (HEK 293T) were obtained from the cell bank of the Chinese Academy of Sciences. HCT116, LOVO, and HEK 293T cells were cultured in Dulbecco’s modified Eagle’s medium (DMEM). DLD1 cells were cultured in RPMI 1640. All were supplemented with 10% fetal bovine serum (FBS), 100 U/mL penicillin and 100 μg/mL streptomycin, and incubated at 37 °C in a humidified incubator with 5% CO_2_. All cell lines were authenticated (Genetica DNA Laboratories). MG-132 (HY-13259, MCE) and Cycloheximide (CHX, HY-12320, MCE) were purchased from MedChemExpress, and dissolved in dimethyl sulfoxide (DMSO) (N182, VICMED) and stored at −80 °C.

Small interfering RNAs against human USP7 and YY1 and nonspecific control siRNAs were procured from GenePharma Technology in Shanghai, China (at a concentration of 50 nM). The respective siRNAs were transfected using jetPRIME® transfection reagent (101000046, Polyplus) according to the manufacturer’s instructions. YY1 and USP7 specific shRNAs were constructed by FulenGen corporation (FulenGen, Guangzhou, China). The sequences of siRNAs and shRNAs were listed in Supplementary methods.

The specific overexpression plasmids of Flag-YY1, HA-USP7, and their corresponding control plasmids were purchased from YouBio (Changsha, Hunan, China). Plasmids of HA-Ub, HA-K48-only Ub, and HA-K63-only Ub and their corresponding control plasmids were purchased from GenePharma Technology in Shanghai, China. Truncated mutants of YY1 were meticulously constructed and authenticated by Genewiz. All the plasmids were transfected using PEI 40 K Transfection Reagent (G1802, Servicebio) according to the manufacturer’s instructions.

### Mass spectrometry (MS) analysis

In this experiment, the isolated YY1 protein was first separated using SDS-PAGE in a 10% acrylamide gel. After separation, the protein bands were stained with Coomassie R-250 and then excised from the gel. To obtain the tryptic peptides, in-gel digestion was performed on the excised gel bands. The tryptic peptides were then subjected to liquid chromatography-tandem mass spectrometry (LC-MS/MS) analysis. To prepare for analysis, the peptides were dissolved in 0.1% formic acid and separated using an EASY-nLC 1000 ultra-performance liquid chromatography (UPLC) system. After separation, the peptides were introduced into the NSI source and analyzed by tandem mass spectrometry (MS/MS) in Q ExactiveTM Plus (Thermo), which was coupled online to the UPLC system. During analysis, electrospray at 2.0 kV was applied, and the scan range was set from 350 to 1800 m/z. Finally, the resulting MS data were processed using Proteome Discoverer 1.3 software, allowing the identification and characterization of the peptides present in the sample.

### Western blot and antibodies

Western blot was performed as previously described [17]. The following antibodies were used in this study for western blot analysis: mouse anti-YY1 (66281-1-Ig, Proteintech), mouse anti-USP7 (66514-1-Ig, Proteintech), mouse anti-glyceraldehyde 3-phosphate dehydrogenase (GAPDH) (60004-1-AP, Proteintech), rabbit anti-flag (20543-1-AP, Proteintech), rabbit anti-HA (51064-2-AP, Proteintech), rabbit anti-Ubiquitin (58395S, Cell Signaling Technology), rabbit anti-LC3B (3868, Cell Signaling Technology) and rabbit anti-TRIAP1 (15351-1-AP, Proteintech).

### Co-immunoprecipitation (CoIP)

Briefly, Protein A/G Magnetic Beads (HY-K0202, MCE) were incubated with the indicated primary antibody 6 h at 4 °C. Subsequently, cell lysis containing an equal amount of protein from control samples and the specific treatments was added to the Protein A/G Magnetic Beads-Antibody complex or anti-Flag magnetic Beads (HY-K0207, MCE). The mixture was then incubated overnight at 4 °C, followed by a collection of the beads were collected and washed four times with binding/wash buffer (1 × PBS + 0.5% Tween-20, pH 7.4). Finally, the beads were boiled in SDS sample buffer and subjected to analysis by immunoblotting.

### Immunofluorescence (IF) assay

In the IF assay, cells were subjected to three consecutive washes using 1 × PBS. Subsequently, these cells were fixed in 4% Paraformaldehyde for a duration of 20 min, followed by a blocking step with 5% bovine serum albumin for 30 min at room temperature. Then, the resultants were incubated with rabbit anti-YY1 (22156-1-AP, Proteintech, 1:100) and mouse anti-USP7 (66514-1-Ig, Proteintech, 1:100). Following another round of washing with 1 × PBS, the samples were incubated with fluorescently-conjugated goat anti-rabbit (SA00013-2, Proteintech) and anti-mouse (SA00013-4, Proteintech) secondary antibodies at a dilution of 1:200 (diluted in 5% BSA) for about 50 min at room temperature. Finally, the cells were washed once more with 1 × PBS before being mounted utilizing an antifade mounting medium containing DAPI (P0131, Beyotime). The acquisition of cell images was carried out using a laser scanning confocal microscope (ZEISS LSM880).

### Molecular docking

The structures of USP7 (Uniprot Q93009) and YY1 (Uniprot P25490) were downloaded from the Uniprot database. The Discovery Studio software was utilized for preprocessing proteins, including the removal of water molecules, adding hydrogen and charges. It also enabled the extraction of the original ligands from the protein structure. Pymol was then employed to visualize the processed protein, and the Zdock module was used to study protein-protein docking.

### RNA extract, reverse transcription-PCR, and qRT-PCR

RNA isolation was performed according to the TRIzol RNA isolation procedure. The complementary DNA (cDNA) was synthesized from 1 μg of RNA using the HiScript First Strand cDNA Synthesis Kit (Vazyme Biotech, Nanjing, China). The UltraSYBR One-Step RT-qPCR Kit (CWBIO, Beijing, China) was used to perform the quantitative real-time polymerase chain reaction (qRT-PCR) analysis. The primers used for qRT-PCR were listed in Supplementary Methods and Table S1.

### RNA-sequencing, ChIP, and PCR

Total RNA was isolated from control or siYY1 LOVO cells, and were sent for RNA-sequencing, which was performed in a Genergy Biotechnology company (Shanghai, China). Chromatin immunoprecipitation (ChIP) assay was performed using the BeyoChIP^TM^ ChIP Assay Kit (Beyotime, China) in HCT116, LOVO, and DLD1 cells according to the manufacturer’s instructions. DNA was purified using a PCR purification kit (Tiangen, China) and prepared for PCR analysis. Then, agarose gel electrophoresis of the PCR product was carried out in 2% agarose gel containing DNA Gel dye. The primers used for ChIP-PCR analysis were listed in Supplementary Methods and Table S2.

### Cell proliferation and colony formation assays

To assess cell proliferation, CCK-8 assay was employed in accordance with the guidelines provided by the manufacturer (Dojindo, Japan). The ability of cells to generate colonies was examined through a colony formation assay. A previous study conducted by our team extensively described the methodology [18].

### Cell migration, invasion, and wound healing assays

Cell migration and invasion were evaluated using the methods of transwell migration assay, wound healing assay, and Matrigel invasion assay. The detailed procedures were on the basis of a previous report [19].

### Xenograft experiments

The Institutional Animal Care and Use Committee at Xuzhou Medical University approved all procedures involving mice, ensuring compliance with ethical regulations. Female BALB/c mice aged 5 to 6 weeks were obtained from GemPharmatech (Nanjing, China) for the Xenograft study. For subcutaneous injection, a suspension of 1 × 10^7^ HCT116 stable cells in 200 μL of PBS with 50% Matrigel (345234, CORNING) was administered to the dorsal flank of each mouse. Mice were randomly divided into four groups: shCtrl, shYY1, shUSP7, and shYY1+shUSP7. Tumor volumes were measured every 2 days in a blinded manner using digital calipers and calculated using the formula V = π/6 (length × width^2^). After 30 days, the mice were euthanized, and the tumors were excised and weighed. Tumor sections were captured, fixed in formalin, and embedded in paraffin. Subsequently, these sections underwent immunohistochemistry (IHC) staining.

### IHC assay

The tissues underwent a series of steps, including deparaffinization and dehydration to prepare them for further analysis. The presence of endogenous peroxidase was blocked by treating the tissues with hydrogen peroxide. Antigen retrieval was performed using either citrate buffer (pH 6.0) or TE buffer (PH 9.0), depending on the product protocol. To prevent nonspecific labeling, the tissues were treated with normal goat serum. Primary antibodies were then applied to the tissues and incubated overnight at 4 °C. Color development was achieved using diaminobenzidine, with hematoxylin used as a counterstain. The following antibodies were used in this study for IHC analysis: rabbit anti-YY1 (22156-1-AP, Proteintech, 1:100), rabbit anti-USP7 (T57219, Abmart, 1:50), rabbit anti-E-cadherin (20874-1-AP, Proteintech, 1:500), rabbit anti-N-cadherin (22018-1-AP, Proteintech, 1:500) and rabbit anti-Ki-67 (27309-1-AP, Proteintech, 1:500).

Two independent investigators evaluated the expression levels of YY1 and USP7. The percentage of tumor cells showing staining was documented using the following scale: 1–24% (scored 1), 25–49% (scored 2), 50–74% (scored 3), and 75–100% (scored 4). Additionally, the intensity of staining was recorded as follows: no staining (scored 0), weak staining (scored 1), moderate staining (scored 2), and strong staining (scored 3). The IHC staining score was calculated by multiplying the percentage of stained tumor cells by the staining intensity. Finally, the IHC results were categorized based on the IHC staining scores.

### Patients and sample collection

The tissue microarrays (TMAs) slides used in this study were obtained from patients with CRC who were enrolled at the Affiliated Hospital of Xuzhou Medical University in China between 2013 and 2015. The TMAs included both CRC tissues and adjacent normal colorectal tissues. The overall survival (OS) time and progression-free survival (PFS) rates were calculated based on the date of surgery to the date of death, tumor progression, or the last follow-up. In addition, tumor tissue slides for correlation analysis of YY1 and USP7 expression were obtained from a cohort of CRC patients enrolled at the Affiliated Hospital of Xuzhou Medical University after September 2020. The tumor budding (TB) grades were also recorded for further analysis. The patient studies were conducted in accordance with the Declaration of Helsinki, and the use of these specimens and data for research purposes was approved by the Ethics Committee of the Hospital.

### Quantification and statistical analysis

The experimental data underwent statistical analysis and were visualized using SPSS V29.0 (SPSS Inc., Chicago, IL, USA) and GraphPad Prism 9.4.1 software (GraphPad Software, USA). T-tests were utilized for comparisons between two groups, and one-way ANOVA with Tukey’s multiple comparison test was used for comparisons among three or more groups. The relationship between YY1 expression and the clinicopathologic parameters was examined using the Chi-square test. To determine the association between YY1 expression and the survival of CRC patients, we utilized the Kaplan–Meier method and the log-rank test. Prognostic risk factors were examined through univariate and multivariate Cox regression analyses. All the experiments were repeated three times independently, with three biological replications per repeat. The data were expressed as the standard error of the mean (SEM), and the mean was represented by a bar. A *P* value of less than 0.05 was considered statistically significant.

## Results

### YY1 was physically associated with USP7

**Fig. 1:**
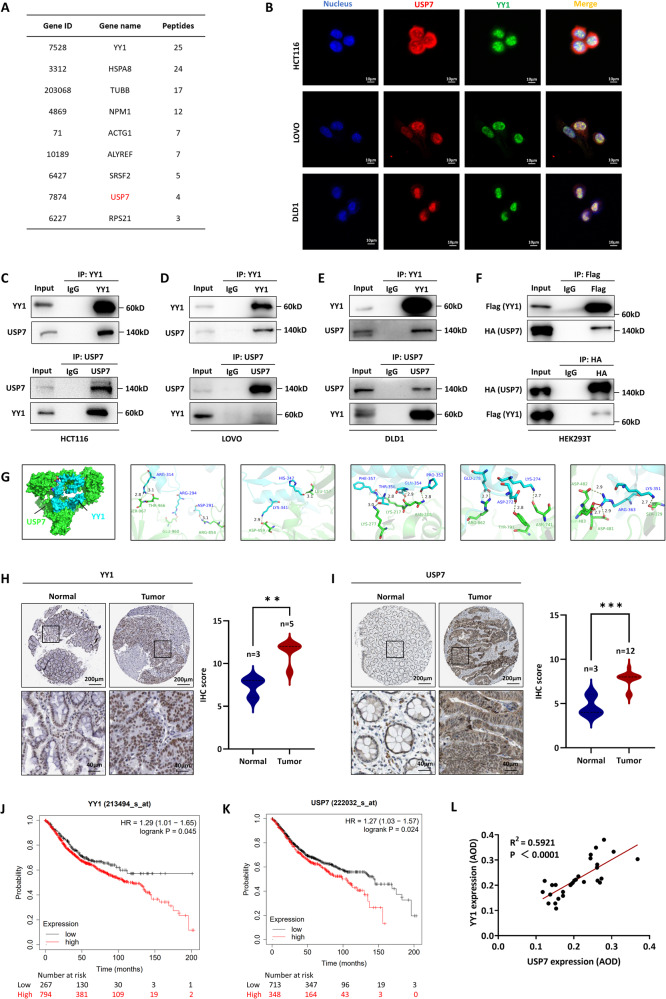
The interaction between YY1 and USP7 and their effect on CRC prognosis. **A** MS analysis to identify that USP7 interacted with YY1. **B** The co-localization of YY1 and USP7 detected by IF assay. **C**–**F** USP7-YY1 interaction in HCT116 (**C**), LOVO (**D**), DLD1 (**E**), and HEK 293T (**F**) cells detected by CoIP. **G** Diagrams of the molecular-docking simulation of YY1 (blue) and USP7 (green) and their interacting amino acid sites. **H**, **I** IHC analysis of YY1 (**H**) and USP7 (**I**) protein expression levels in tumors and normal colorectal tissues. Data were obtained from HPA. **J**, **K** Overall survival analysis of YY1 (**J**) and USP7 (**K**) in CRC clinical data from Kaplan–Meier Plotter. **L** YY1 and USP7 expression levels in CRC patients were positively correlated. Data were obtained from the tumor tissue slides of 30 CRC patients. ^*^P < 0.05, ^**^P < 0.01.

To gain a comprehensive understanding of YY1’s function, the interaction proteins of YY1 were identified by conducting affinity purification and MS analysis in whole-cell extracts from HCT116 cells expressing Flag-YY1 (Fig. 1A). Various protein interactions were found to be associated with YY1, including the presence of USP7 in the YY1-containing protein complex. In order to investigate the correlation between YY1 and USP7, IF experiments were carried out in three CRC cell lines (HCT116, LOVO, and DLD1). The findings exhibited spatial co-localization of YY1 and USP7 in all three CRC cell lines (Fig. 1B). This was further supported by CoIP experiments, in which YY1 efficiently co-immunoprecipitated with USP7, and vice versa, in CRC cells (Fig. 1C–E). The same observations were made in HEK 293T cell extracts with exogenous Flag-YY1 and HA-USP7 (Fig. 1F). Additionally, computer simulations were employed to evaluate the protein-protein interaction interface of USP7 and YY1. The analysis indicated that USP7 and YY1 were predicted to interact with each other through salt bridge (e.g., GLU960/ARG294, ASP481/ARG363) and hydrogen bond (e.g., LEU157/HIS347, SER129/LYS351, and ASP459/LYS341) (Fig. 1G). These results reveal the physical interaction between YY1 and USP7.

### Both USP7 and YY1 influenced CRC prognosis

To understand the functional significance of the physical interaction between USP7 and YY1, clinical data were analyzed to investigate their impact on CRC. Analysis of data from the HPA database (http://www.proteinatlas.org) revealed elevated expression levels of both USP7 and YY1 in CRC tissues compared to normal colorectal tissues (Fig. 1H, I). Survival analysis using the Kaplan–Meier Plotter (http://kmplot.com) also demonstrated that patients with higher expression of USP7 and YY1 showed a worse prognosis in colon cancer (Fig. 1J, K). To further investigate this association, tumor tissue slides from 30 CRC patients were examined. The analysis showed a positive correlation between the expression levels of YY1 and USP7 in these patients (Fig. 1L).

These findings collectively indicate the presence of an interaction between USP7 and YY1 in CRC cells, and their potential involvement in the malignant progression of CRC. Therefore, further study is needed to explore the underlying mechanisms of this interaction.

### USP7 stabilized YY1 through its deubiquitylation activity

**Fig. 2:**
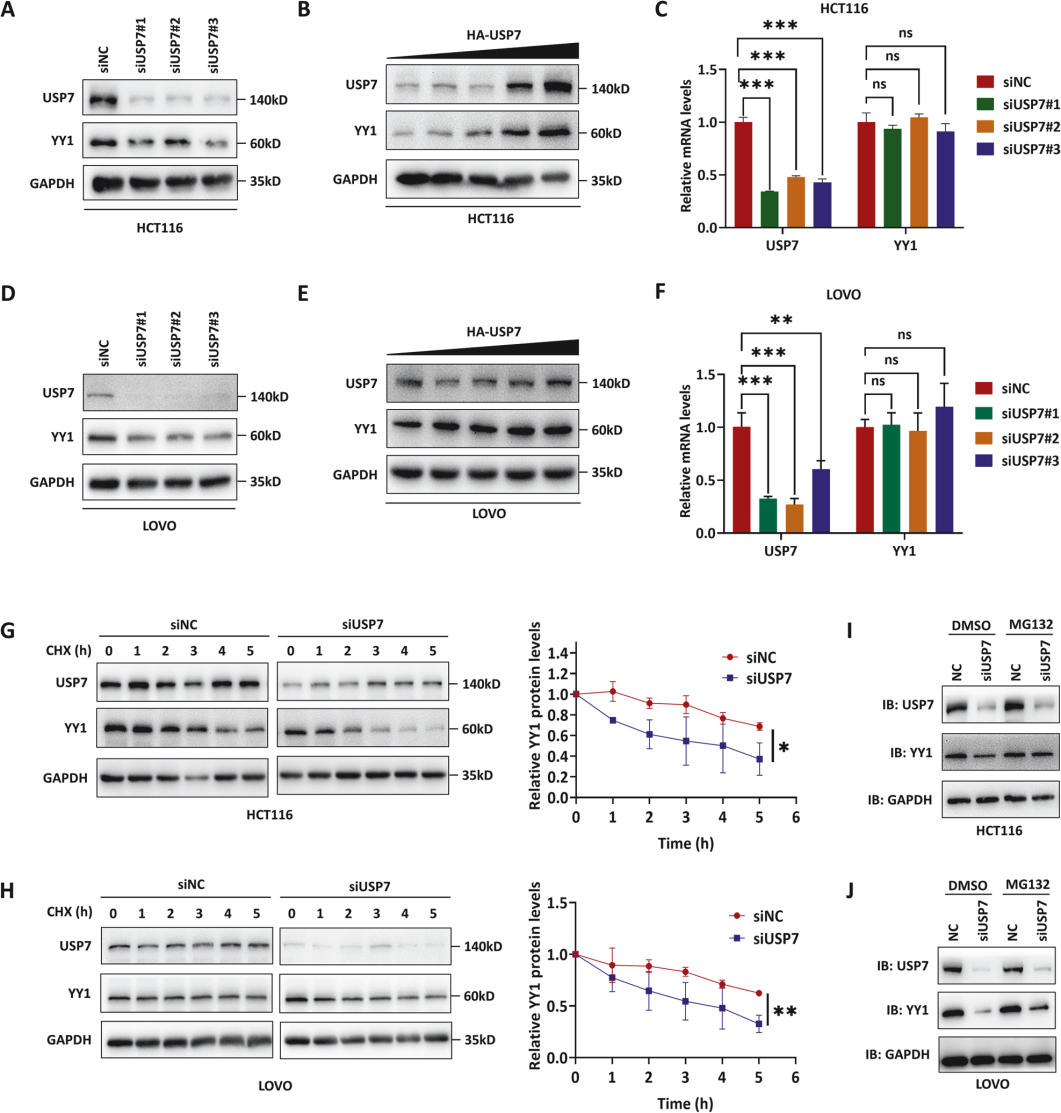
USP7 stabilized YY1 protein expression. **A**–**C** The expressions of YY1 and USP7 at mRNA and protein levels were detected by qPCR and western blot with corresponding treatment in HCT116 cells. **D**–**F** The expressions of YY1 and USP7 at mRNA and protein levels were detected by qPCR and western blot with corresponding treatment in LOVO cells. **G**, **H** Effect of siUSP7 on YY1 degradation in HCT116 (**G**) and LOVO (**H**) cells transfected with siUSP7 and treated with CHX. I, G Effect of USP7 on YY1 degradation in HCT116 (**I**) and LOVO (**J**) cells transfected with siUSP7 and treated with MG-132. Data were expressed as the mean ± SD, ns, no significance,*P < 0.05, **P < 0.01, ***P < 0.001.

SiUSP7 or siYY1 RNA was transfected into HCT116 and LOVO cells to verify the interaction between USP7 and YY1 and their reciprocal effect. Western blot analysis showed that the downregulation of USP7 reduced the protein expression level of YY1 (Fig. 2A, D). Consistently, USP7 overexpression led to an increase of YY1 protein expression (Fig. 2B, E). On the other hand, there was no significant influence on YY1 mRNA level when USP7 was knocked down according to the qRT-PCR analysis (Fig. 2C, F). Cycloheximide (CHX), the protein synthesis inhibitor, was used to detect the half-life of YY1 in HCT116 and LOVO cells with diminished levels of USP7. The results revealed a reduction in the lifespan of YY1 within the siUSP7-treated group (Fig. 2G, H). The application of the proteasome inhibitor MG-132 on USP7 knockdown CRC cells further revealed that the reduction in YY1 protein expression resulting from USP7 knockdown can be partially reversed by inhibiting the proteasome degradation pathway (Fig. 2I, J). These findings suggest that USP7 plays a role in stabilizing YY1 and preventing its degradation through the proteasome pathway.

**Fig. 3:**
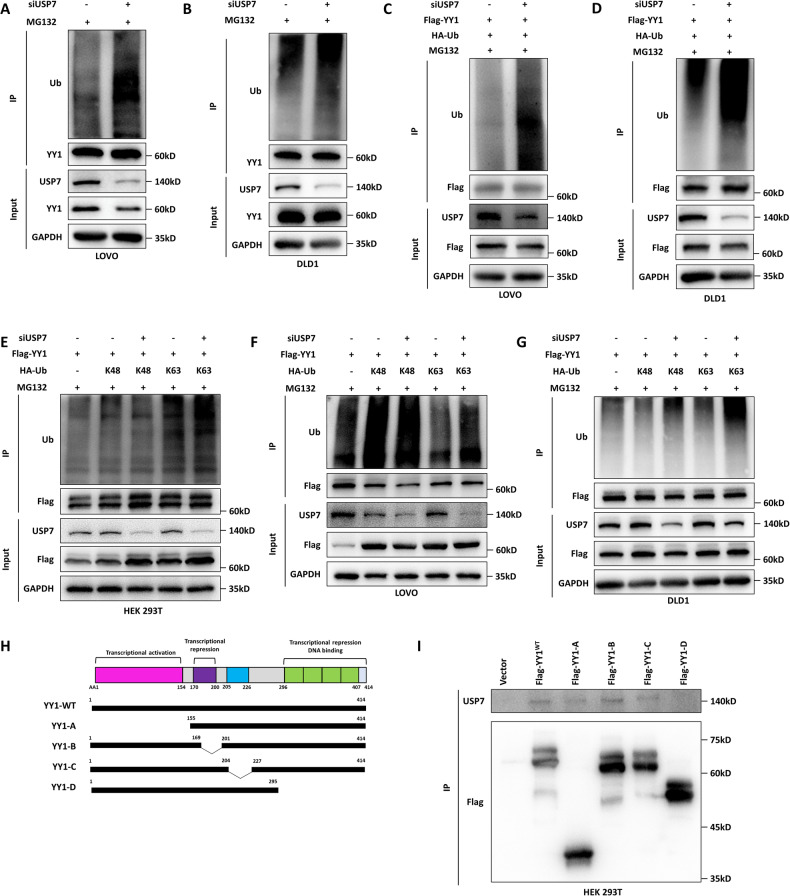
USP7 promoted YY1 stabilization by deubiquitinating it through the K63 linkage. **A**, **B** The effect of USP7 on YY1 ubiquitination in LOVO (**A**) and DLD1 (**B**) cells treated with siUSP7. **C**, **D** The effect of USP7 on YY1 ubiquitination in LOVO (**C**) and DLD1 (**D**) cells treated with siUSP7 followed by transfection of Flag-YY1 and HA-Ub. **E**–**G** K48-only and K63-only HA-Ub plasmids alone or cotransfected with siUSP7 into HEK 293T, LOVO, and DLD1 cells to detect YY1 ubiquitination by CoIP. **H** Schematic representation of YY1 truncated mutaions. **I** The YY1 truncated mutation plasmids were transfected into HEK 293T to detect their interaction with USP7.

The role of USP7 as a deubiquitinating enzyme led to the hypothesis that it could affect the stability of YY1 by deubiquitylation. When USP7 was knocked down, it was observed that the levels of ubiquitinated YY1 protein significantly increased (Fig. 3A–D and Supplementary Fig. 1A, B). Further investigation revealed that siUSP7 mainly induced elevated ubiquitination of YY1 via the K63 linkage (Fig. 3E–G and Supplementary Fig. 1C). In order to identify the specific domain of YY1 that interacts with USP7, truncated mutants of YY1 were created (Fig. 3H). The results indicated that the mutant lacking the 296–414 amino acid residue showed impaired interactions with USP7, suggesting that this particular domain plays a crucial role in the interaction between USP7 and YY1 (Fig. 3I). Overall, these findings confirm that USP7 promotes YY1 stabilization by deubiquitinating it through the K63 linkage.

### YY1 transcriptionally regulated TRIAP1 and LC3B expressions

**Fig. 4:**
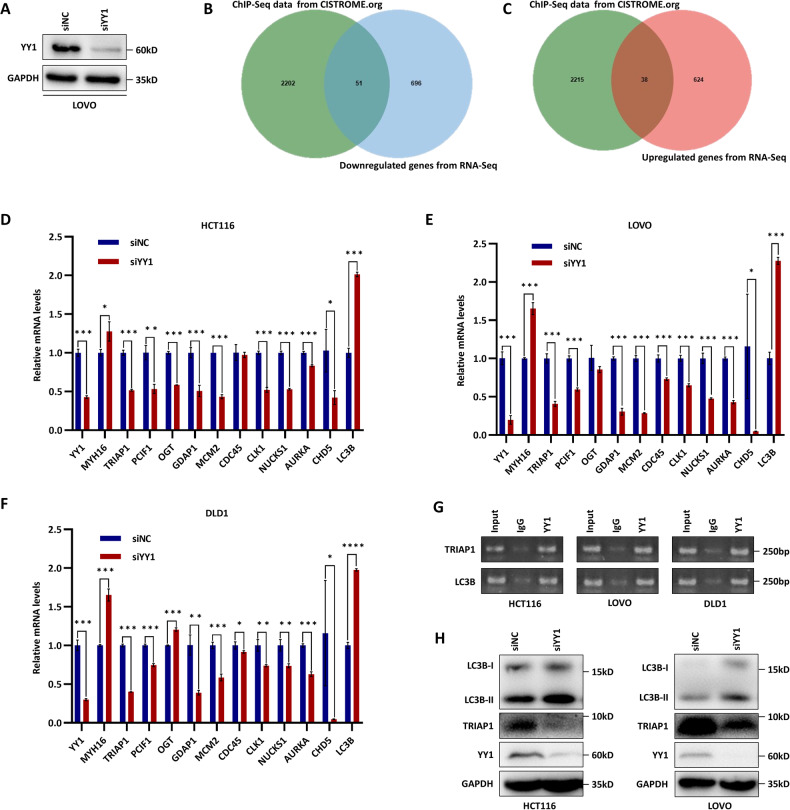
YY1 transcriptionally regulated TRIAP1 and LC3B expressions. **A** Western blot verification of siYY1 efficiency in LOVO cells for further RNA-sequencing. **B**, **C** Overlap of differentially expressed genes identified by RNA-sequencing and ChIP-sequencing data from CISTROME.org. **D**–**F** Validation of RNA-sequencing data in HCT116 (**D**), LOVO (**E**), and DLD1 (**F**) cells using qRT-PCR. **G**, **H** Validation of RNA-sequencing data using ChIP (**G**) and western blot (**H**) assays. Data were expressed as the mean ± SD, *P < 0.05, **P < 0.01, ***P < 0.001.

To explore the downstream pathways of the USP7-YY1 interaction network, we performed RNA-sequencing analysis in LOVO cells following YY1 knockdown (Fig. 4A), which was integrated with the ChIP-sequencing data from the CISTROME Data Browser. Comparative analysis revealed a remarkable overlap of 51 genes that were downregulated and 38 that were upregulated (Fig. 4B, C). Subsequent extensive literature review led to the identification of ten downregulated genes and two upregulated genes that have been implicated in tumor development, including but not limited to CRC.

qRT-PCR analysis was performed to validate the expression changes of a subset of eight genes in HCT116, LOVO, and DLD1 cells with YY1 knockdown (Fig. 4D–F). Additionally, the ChIP-PCR assay confirmed that YY1 is bound to the promoters of TRIAP1 and LC3B (Fig. 4G). Western blot experiments provided further validation, showing a decrease in TRIAP1 protein expression and an increase in LC3B expression following YY1 knockdown (Fig. 4H). Previous studies have suggested that TRIAP1 functions as a tumor-promoting gene [20–24], whereas LC3B acts as a tumor suppressor gene [25–27]. The findings are consistent with the role of YY1 in promoting tumor development through the repression of tumor suppressor genes and activation of tumor-promoting genes, as YY1 performs bidirectional transcriptional regulation depending on the context.

### YY1 and USP7 promoted tumorigenesis and metastasis of colorectal carcinoma in vitro and in vivo

**Fig. 5:**
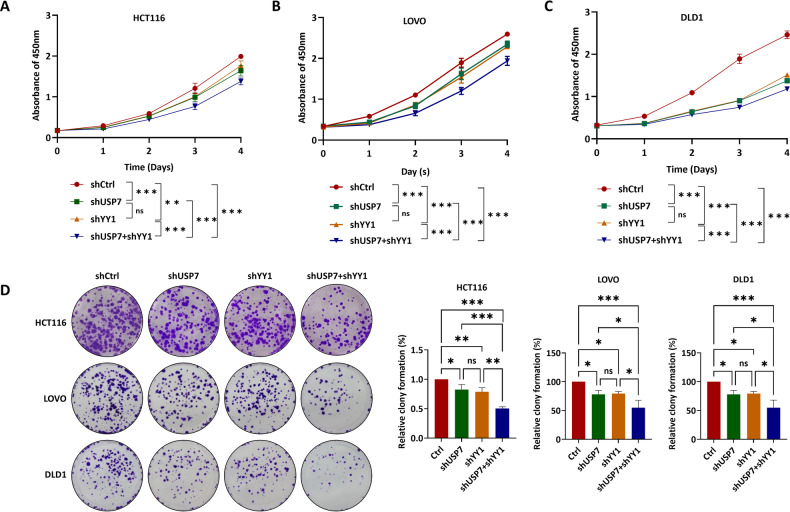
YY1 and USP7 promoted the proliferation of CRC in vitro. **A**–**C** CCK-8 assays were used to assess the effect of USP7, YY1, or both deficiency on cell proliferation in HCT116 (**A**), LOVO (**B**), and DLD1 (**C**) cells. **D** Clone formation assays for USP7, YY1, or both deficiency in HCT116, LOVO, and DLD1 cell lines. Data were expressed as the mean ± SD (ns, no significane, *P < 0.05, **P < 0.01, ***P < 0.001).

**Fig. 6:**
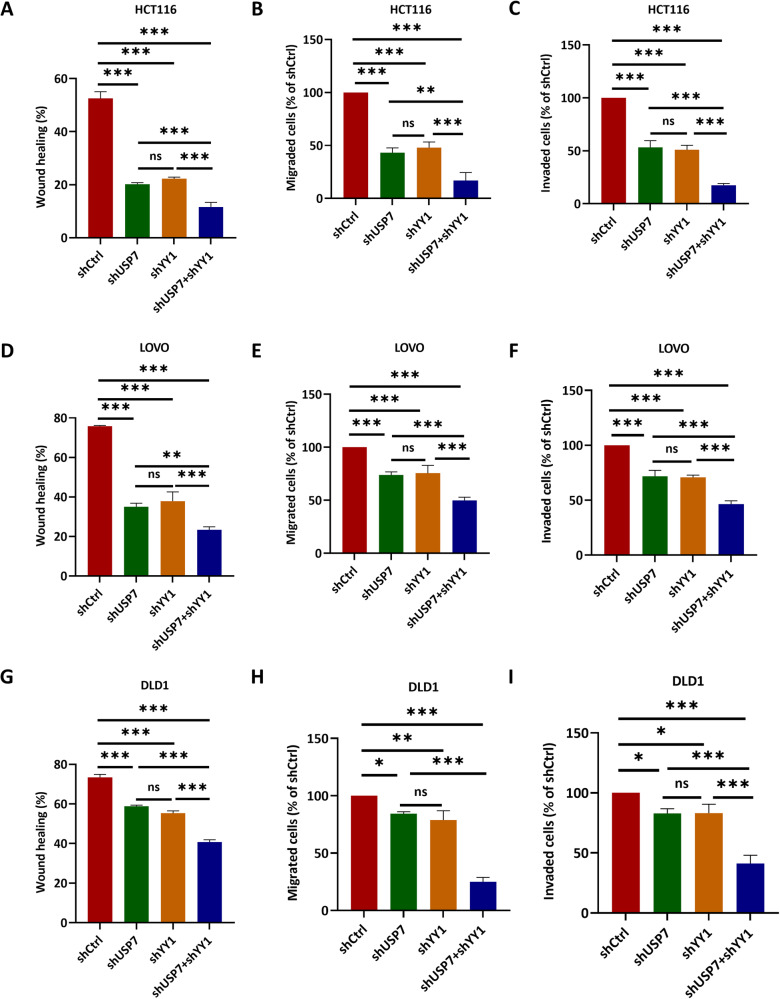
YY1 and USP7 promoted migration and invasion of CRC in vitro. Wound healing, transwell migration, and Matrigel invasion assays were used to detect the migration and invasion ability of HCT116 (**A**–**C**), LOVO (**D**–**F**), and DLD1 (**G**–**I**) cells with USP7, YY1 or both deficiency. Data were expressed as the mean ± SD, ns no significane, ^*^P < 0.05, ^**^P < 0.01, ^***^P < 0.001.

To investigate the biological function of USP7-mediated YY1 stabilization in CRC, we established stable cell lines with knockdown of YY1, USP7, or both using lentivirus infection. Our findings showed that knocking down YY1 or USP7 individually decreased the viability of CRC cells to a similar extent. However, double knockdown further attenuated the proliferation of CRC cells, as demonstrated by both cell proliferation assays (Fig. 5A–C) and clone formation assays (Fig. 5D). Wound healing and transwell assays revealed that knockdown of USP7 or YY1 inhibited cell migration and invasion, and double knockdown enhanced this suppression effect in HCT116 (Fig. 6A–C), LOVO (Fig. 6D–F), and DLD1 cells (Fig. 6G–I).

**Fig. 7:**
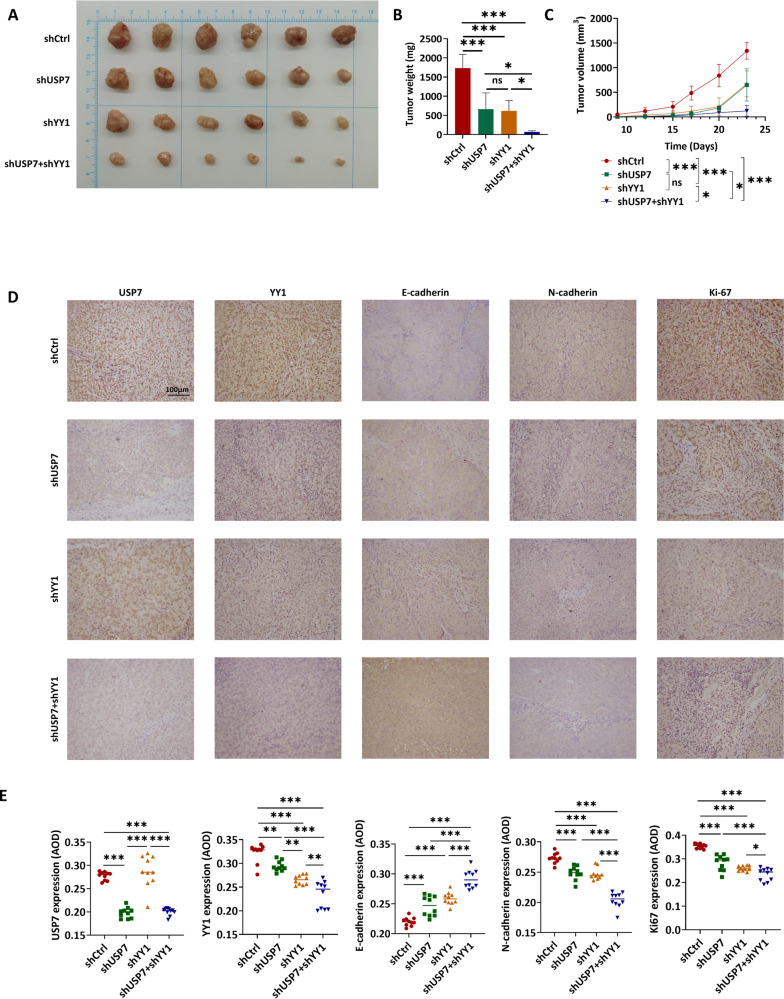
YY1 and USP7 promoted tumorigenesis and metastasis of colorectal carcinoma in vivo. **A**–**C** Tumor tissue images (**A**), tumor weight (**B**), and tumor volume statistical curves (**C**) of shCtrl, shUSP7, shYY1 and shUSP7+shYY1 HCT116 tumor-bearing mice. **D** Representative IHC staining images of USP7, YY1, E-cadherin, N-cadherin, and Ki-67 in tumor tissue sections of each group. **E** The statistic analysis of IHC staining in (**D**). Data were expressed as the mean ± SD, ns no significane, ^*^P < 0.05, ^**^P < 0.01, ^***^P < 0.001.

Moreover, we established xenograft tumor models by subcutaneously transplanting HCT116 cells with stable knockdown of USP7, YY1, or both into BALB/c nude mice. Tumor growth was assessed based on the weight and volume, revealing a significant inhibition of tumor growth in cases of YY1 or USP7 downregulation. The shYY1+shUSP7 group exhibited the lowest tumor growth (Fig. 7A–C). IHC staining of tumor tissues revealed that USP7 expression was reduced in the shUSP7 and shUSP7+shYY1 groups, but showed no significant change in the shYY1 group. YY1, N-cadherin, and Ki-67 expressions decreased in two single knockdown groups and further decreased in the double knockdown group. In contrast, E-cadherin expression increased in the shUSP7 and shYY1 groups, reaching the highest level in the shUSP7+shYY1 group (Fig. 7D, E). These results imply that the expression of YY1 could be decreased by USP7 knockdown, consequently leading to the inhibition of CRC cell proliferation, migration, invasion, and epithelial-mesenchymal transition (EMT).

### YY1 was overexpressed in patients with CRC and was associated with poor outcome

**Fig. 8:**
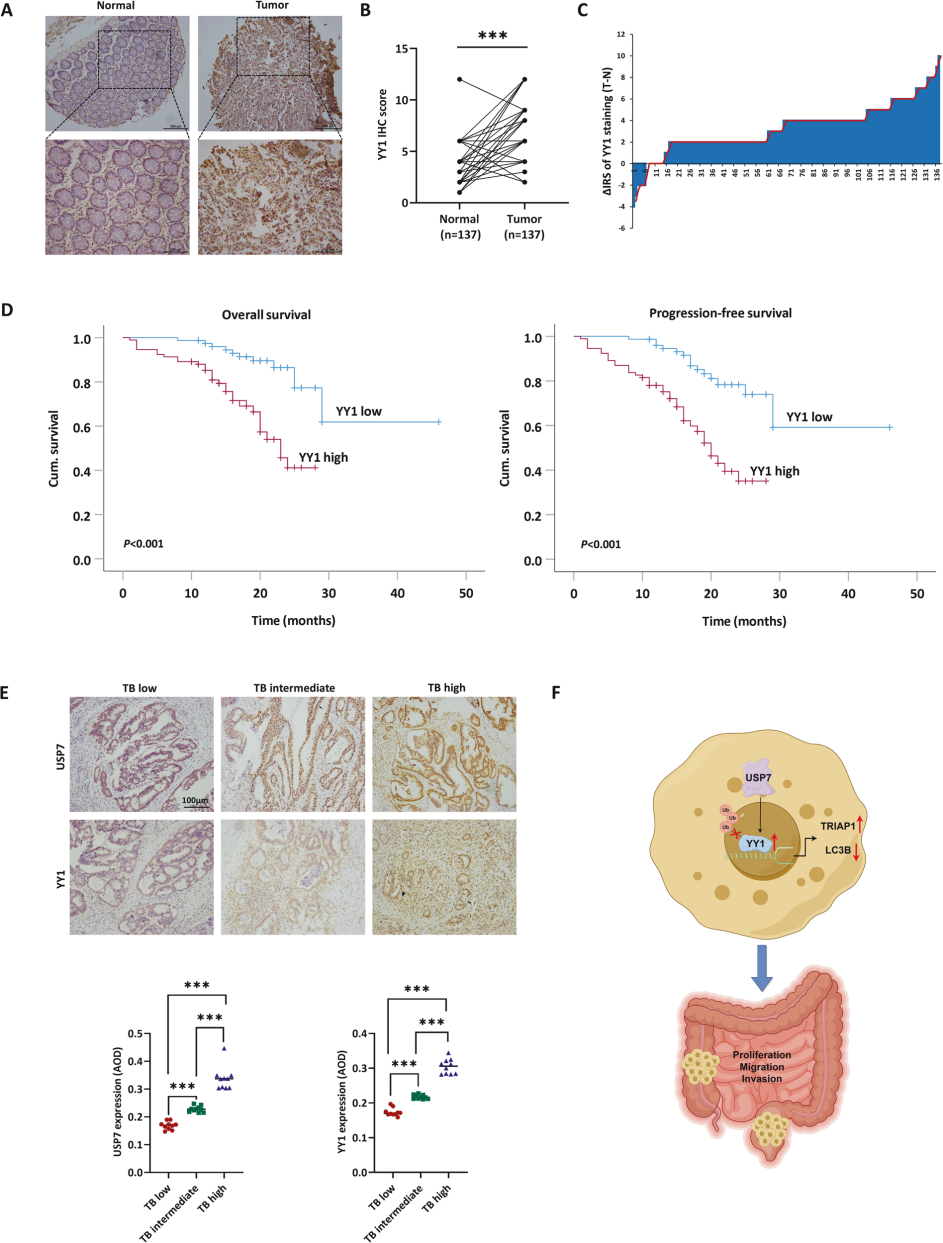
YY1 was overexpressed in patients with CRC and was associated with poor outcomes. **A**, **B** Representative IHC staining images (**A**) and IHC score (**B**) of YY1 protein expression in 137 paired adjacent non-cancerous tissue and CRC tissues. **C** Staining intensities of YY1 in CRC tissues compared with paired adjacent non-cancerous tissue. (N, paired adjacent non-cancerous tissues. T, tumor tissues) **D** Kaplan–Meier survival curves depicting OS or PFS (*n* = 188, *p* < 0.001). **E** Representative IHC staining images and staining intensity statistics of USP7 and YY1 with different TB grades. **F** Schematic diagram of the interaction between USP7 and YY1 by Figdraw. Data were expressed as the mean ± SD, ^***^P < 0.001.

To investigate the impact of YY1 in the development of CRC, a study using tissue microarray (TMA) slides from CRC patients was conducted to analyze the expression of YY1 protein. The results showed a significant upregulation of YY1 expression in cancerous tissue compared to their adjacent non-cancerous counterparts in CRC patients (Fig. 8A–C). We also examined the correlation between YY1 expression and the clinicopathologic characteristics in 137 pairs of CRC tissue samples. It was found that high levels of YY1 expression were associated with poor differentiation (*P* <0.001), positive lymph node metastasis (*P* = 0.014), and advanced TNM stage (*P* = 0.014) in CRC patients (Table 1). Moreover, Kaplan–Meier survival analysis of 188 CRC patients showed that high YY1 levels were significantly associated with poor OS (*P* < 0.001) and PFS (*P* < 0.001) (Fig. 8D). To determine whether YY1 expression acted as an independent prognostic factor for CRC, univariate and multivariate Cox regression analysis models were utilized. The univariate analysis suggested that YY1 expression held substantial prognostic significance for OS and PFS in CRC patients (Table 2). Importantly, the multivariate analysis unveiled that YY1 expression served as an independent prognostic marker for both OS (HR 2.419, 95% CI 1.102 to 5.311, *P* = 0.028) and PFS (HR 2.256, 95% CI 1.152 to 4.419, *P* = 0.018) in CRC patients (Table 3).

**Table 1:** Relationship between YY1 expression and clinicopathological features of CRC patients.

Variables	YY1 staining			
	Low (%)	High (%)	Total	P^*^
Age				
≤55 years	23 (41.1)	33 (58.9)	56	0.160
>55 years	69 (52.3)	63 (47.7)	132	
Gender				
Male	54 (51.4)	51 (48.6)	105	0.442
Female	38 (45.8)	45 (54.2)	83	
Location				
Colon	48 (53.9)	41 (46.1)	89	0.194
Rectum	44 (44.4)	55 (55.6)	99	
Tumor diameter				
≤5 cm	68 (52.3)	62 (47.7)	130	0.166
>5 cm	24 (41.4)	34 (58.6)	58	
Differentiation				
Poor	11 (22.0)	39 (58.7)	50	<0.001
Moderate/high	81 (78.0)	57 (41.3)	138	
Lymph node metastasis				
Negative	64 (56.1)	50 (43.9)	114	0.014
Positive	28 (37.8)	46 (62.2)	74	
Distance metastasis				
Negative	88 (48.9)	92 (51.1)	180	1.000
Positive	4 (50.0)	4 (50.0)	8	
TNM stage				
I/II	64 (56.1)	50 (43.9)	114	0.014
III/IV	28 (37.8)	46 (62.2)	74	

*P values are from *χ*^2^ test.

**Table 2:** Univariate Cox regression analysis on overall survival and progression-free survival of colorectal cancer patients.

Variables	Overall survival			Progression-free survival		
	Hazard ratio	95% CI^#^	P^*^	Hazard ratio	95% CI^#^	P^*^
YY1						
Low	1.000		<0.001	1.000		<0.001
High	4.520	2.170–9.413		4.020	2.154–7.500	
Age						
≤55 years	1.000		0.179	1.000		0.015
>55 years	0.648	0.344–1.220		0.506	0.293–0.876	
Gender						
Male	1.000		0.460	1.000		0.303
Female	1.264	0.679–2.353		1.332	0.772–2.297	
Location						
Colon	1.000		0.877	1.000		0.886
Rectum	1.050	0.565–1.952		0.961	0.558–1.654	
Tumor diameter						
≤5 cm	1.000		0.960	1.000		0.962
>5 cm	0.983	0.499–1.933		1.014	0.563–1.829	
Differentiation						
Poor	1.000		<0.001	1.000		<0.001
Moderate/high	0.147	0.076–0.284		0.217	0.126–0.374	
Lymph node metastasis						
Negative	1.000		<0.001	1.000		<0.001
Positive	4.048	2.093–7.829		5.631	3.091–10.258	
Distant metastasis						
Negative	1.000		0.014	1.000		0.005
Positive	3.706	1.304–10.532		3.783	1.489–9.614	
TNM stage						
I/II	1.000		<0.001	1.000		<0.001
III/IV	4.048	2.093–7.829		5.631	3.091–10.258	

*P values are from the log-rank test.

^#^CI: confidence interval.

**Table 3:** Multivariate Cox regression analysis on overall survival and progression-free survival of colorectal cancer patients.

Variables	Overall survival			Progression-free survival		
	Hazard ratio	95% CI^#^	P^*^	Hazard ratio	95% CI^#^	P^*^
YY1						
Low	1.000		0.028	1.000		0.018
High	2.419	1.102–5.311		2.256	1.152–4.419	
Age						
≤55 years	1.000		0.583	1.000		0.580
>55 years	1.200	0.626–2.299		0.854	0.487–1.476	
Differentiation						
Poor	1.000		<0.001	1.000		<0.001
Moderate/high	0.217	0.106–0.445		0.369	0.204–0.667	
Distant metastasis						
Negative	1.000		0.039	1.000		0.157
Positive	3.277	1.063–10.101		2.027	0.762–5.391	
TNM stage						
I/II	1.000		0.016	1.000		<0.001
III/IV	2.376	1.175–4.803		3.968	2.106–7.476	

^*^P values are from the log-rank test.

^#^CI: confidence interval.

Additionally, we conducted a measurement of YY1 expression levels in patients exhibiting varying TB grades. TB refers to the presence of dedifferentiated tumor cells, which can manifest as solitary cells or small clusters containing up to four cells located at the invasive frontier of diverse cancers [28]. It is well-documented that TB is linked to EMT and reduced survival rates. Recently, TB has gained recognition as a crucial prognostic indicator for recurrence among CRC patients, leading to its incorporation into routine postoperative pathological assessments [29, 30]. In order to delve deeper into this phenomenon, we compiled a cohort comprising 30 CRC patients encompassing varying TB grades (with an equal distribution of 10 patients per grade) and evaluated the expression of YY1 within their tumor tissues. Our findings conclusively revealed a noteworthy elevation in YY1 expression levels as TB grade increased, strongly implying a positive correlation between YY1 expression and the recurrence of tumors (Fig. 8E).

## Discussion

YY1, an extensively studied transcriptional regulator, has garnered significant attention due to its ubiquitous expression, highly conserved molecular structure, and pivotal role in various aspects of embryonic development and cellular activities, such as DNA replication, cell growth, aging, and response to DNA-damaging agents [31, 32]. Increased expression or activation of YY1 is linked to unregulated cell proliferation, resistance to programmed cell death, the formation of tumors, and the potential for metastasis [33]. The overwhelming body of evidence suggests that YY1 predominantly functions to facilitate tumor development in various cancers [6]. Due to its multifaceted functions enabling context-dependent and contradictory outcomes in transcriptional regulation (such as initiation, activation, and repression), it is of importance to fully understand the regulatory mechanisms of YY1 expression and its downstream target genes in cancer context.

In this study, we discovered the interaction between YY1 and USP7, a member of the deubiquitinating enzyme family. The clinical data analysis with various public databases and CRC tumor tissues confirmed the high expression levels of USP7 and YY1 in CRC, along with a positive correlation between them. Moreover, both the higher expressions of USP7 and YY1 implied an unfavorable prognosis for CRC patients. To elaborate further, USP7 acted as a key player in modulating the protein level of YY1 in CRC. By binding to the 296–414 amino acid residues of YY1, USP7 attenuates its K63-linked ubiquitination and degradation, resulting in the prolonged functional lifespan of YY1. Further RNA-sequencing and bioinformatic analysis screened out 12 possible downstream target genes. qRT-PCR, ChIP, and western bot assays finally identified two candidates, TRIAP1 and LC3B.

TP53-regulated apoptosis inhibitor 1(referred to as TRIAP1 or P53CVs), upregulated by TP53 in response to low genotoxicity, is a necessary protein for the maintenance of mitochondrial morphology and is mainly involved in the cellular survival pathways regulated by TP53 [20]. Mechanistically, TRIAP1 interacts with HSP70, activates caspase-9, and inhibits the interaction between cytochrome C and the apoptotic protease activating factor 1, resulting in increased resistance to apoptosis and allows for efficient DNA damage repair [34, 35]. Numerous studies consistently demonstrate high expression levels of TRIAP1 in tumor cells, thereby facilitating the progression of various malignant tumors [20–24]. Furthermore, its elevated expression in cancer cells is strongly associated with drug resistance [35, 36].

LC3B (Microtubule-associated proteins 1A/1B light chain 3B, MAP1LC3B) is the coding gene for LC3, which is the central protein and critical biomarker in the autophagy pathway [37, 38]. The role of LC3B in tumorigenesis varies throughout different stages of tumor development due to the dynamic nature of the autophagic flux [39, 40]. Nuta et al. identified the LC3B^Y113C^ mutation predominantly in primary tumors, suggesting a potential tumor-promoting effect by impairing cellular autophagic capacity. The reduced levels of autophagy lead to increased reactive oxygen species and DNA damage, thereby promoting cancer at the early stage [25]. Meanwhile, the positivity of LC3B+ puncta in breast cancer cells is positively correlated with the presence of intratumoral CD8 + T lymphocytes but negatively correlated with the presence of immunosuppressive cell types, resulting in enhanced anti-tumor immune microenvironment and better long-term survival [26, 27].

Our study found that YY1 could bind to the promoter regions of TRIAP1 and LC3B to transcriptionally activate TRIAP1 but suppress LC3B. Consistently, YY1 knowdown decreased the expression of TRIAP1 but enhanced the expression of LC3B in both mRNA and protein levels. Therefore, the activation of TRIAP1 and suppression of LC3B might contribute to the tumor-promoting function of YY1 in CRC.

The significance of YY1 in oncogenic pathways has sparked extensive research efforts aimed at inhibiting YY1 through various direct and indirect targets for therapeutic purposes [41]. The reported strategies for inducing YY1 inhibition include direct phosphorylation, increasing ubiquitination, microRNA regulation, betulinic acid, and NO donors [42, 43]. Additionally, a small synthetic inhibitor of YY1 (Inh-YY1) is currently in development, offering promise in addressing the need for a specific YY1 inhibitor [41]. On the other hand, several USP7 inhibitors have been identified, showing encouraging results in vitro and in vivo studies. However, none of these compounds have progressed to clinical trials, possibly due to limitations such as lack of selectivity, low potency, potential toxicity, solubility issues, and limited data on pharmacokinetics [10, 44, 45]. Therefore, investigating the interplay between USP7 and YY1 could help in developing YY1 inhibitors and addressing obstacles in USP7 inhibitor research. Furthermore, the combined targeting of both proteins has shown enhanced anti-tumor effects in preclinical models, while potentially minimizing toxicity, offering a promising dual-target strategy for cancer therapy.

However, our study only focused on the interaction between USP7 and YY1, without delving into the detailed degradation pathways of YY1. To date, only a single literature report mentioned that USP7 may stabilize YY1 protein by inhibiting its ubiquitin-mediated proteasomal degradation, which is consistent with the results of our study [46]. Yet, as a ubiquitously distributed transcriptional factor, YY1 is stably expressed in the nucleus, while the degradation process requires it to move from the nucleus to the cytoplasm. The dynamic subcellular distribution of YY1 was previously reported by Palko and colleagues [47]. Their findings showed that YY1 is mainly located in the cytoplasm at the G1 phase, transitions to the nucleus at the early and mid-S phase, and then relocates back to the cytoplasm at the late S phase. USP7 inhibitors have been reported to induce DNA damage and cell-cycle arrest in a p53-dependent or p53-independent manner [48, 49]. Therefore, we reasonably speculate that USP7 may stabilize YY1 by regulating the cell-cycle process. Future research will focus on the nucleocytoplasmic shuttling of YY1 and its final whereabouts.

In summary, our data have presented that USP7 stabilizes the expression of YY1 via interfering its K63-linked ubiquitination and prostasome-mediated degradation. YY1 subsequently activates TRIAP1 but inactivates LC3B, thereby promoting the development of CRC (Fig. 8F). By establishing the interaction between YY1 and USP7, our study provides a valuable foundation for further research into the precise mechanisms and functions related to this interaction. Understanding the intricate interplay between YY1 and USP7 could potentially uncover novel therapeutic targets or predictive markers for various diseases, as both proteins are known to be involved in a wide range of cellular activities.

### Supplementary information

Supplementary figure 1

Supplementary Methods

Raw data of Western blotting

## Data Availability

Data and materials supporting the findings of this study are available from the corresponding author upon reasonable request.
