# Supplementary material for: The role of USP7-YY1 interaction in promoting colorectal cancer growth and metastasis
Source: Cell Death Dis. 2024 May 20;15(5):347. doi: 10.1038/s41419-024-06740-4 (PMC11106261; doi:10.1038/s41419-024-06740-4)
Supplement: Supplementary file 1 — Supplementary figure 1 [file 41419_2024_6740_MOESM1_ESM.docx]

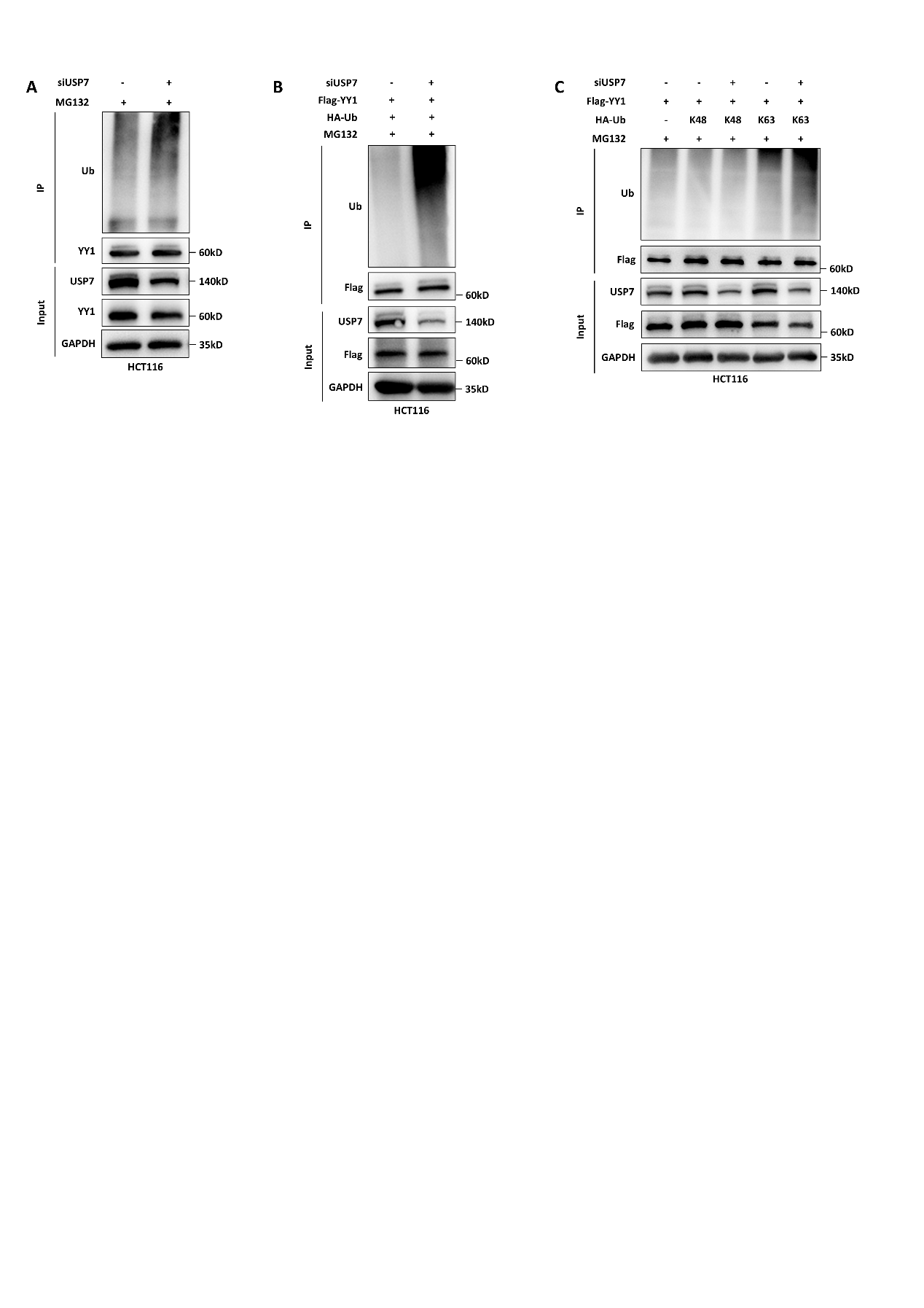
**Supplementary Figure 1. Ubiquitinaition expriments in HCT116 cells. A** The effect of USP7 on YY1 ubiquitination in HCT116 cells treated with siUSP7. **B** The effect of USP7 on YY1 ubiquitination in HCT116 cells treated with siUSP7 followed by transfaction of Flag-YY1 and HA-Ub. **C** K48-only and K63-only HA-Ub plasmids alone or cotransfected with siUSP7 into HCT116 cells to detect YY1 ubiquitination by CoIP.
