# Supplementary material for: The role of USP7-YY1 interaction in promoting colorectal cancer growth and metastasis
Source: Cell Death Dis. 2024 May 20;15(5):347. doi: 10.1038/s41419-024-06740-4 (PMC11106261; doi:10.1038/s41419-024-06740-4)
Supplement: Supplementary file 2 — Supplementary Methods [file 41419_2024_6740_MOESM2_ESM.docx]

**Supplementary Methods**

**The siRNAs sequences used in the study**

siUSP7#1: GCAGUGCUGAAGAUAAUAATT;

siUSP7#2: GUGGUUACGUUAUCAAAUATT;

siUSP7#3: GCCCGGUAAUAUGUCUCAUTT;

siYY1: CCAUGAGACAGUGGUUGAATT;

SiNC: UUCUCCGAACGUGUCACGUTT.

**The shRNAs sequences used in the study**

shCtrl: GCTTCGCGCCGTAGTCTTA

shUSP7#1: CCAGCAATGTTAGATAATGAA

shUSP7#2: GCAATGCTGAATCTGATTCCA

shUSP7#3: CCAGTGTAAAGAAGTAGACTA

shYY1#1: ACGGCTTCGAGGATCAGATTC

shYY1#2: TTGGAGAGAACTCACCTCCTG

shYY1#3: ACATCTTAACACATGCTAAGG

**Table S1.** The primers for qRT-PCR analysis.

|  | **Forward** | **Reverse** |
| --- | --- | --- |
| ***GAPDH*** | AAGGTCGGAGTCAACGGATTTG | CCATGGGTGGAATCATATTGGAA |
| ***YY1*** | GGAGGAATACCTGGCATTGACC | CCCTGAACATCTTTGTGCAGCC |
| ***USP7*** | GTCACGATGACGACCTGTCTGT | GTAATCGCTCCACCAACTGCTG |
| ***CHD5*** | CGAAGGCTACAAGTATGAGCGG | GGTTGAGAGGAGGAAGCAGAAC |
| ***MYH16*** | TCCAGGCAGAAGTTGAGGACCT | GCAACTCCTCACACTTCTGCTG |
| ***TRIAP1*** | CGCTGGTTCGCCGAGAAATTTC | GCCCATGAACTCCAGTCCTTCA |
| ***PCIF1*** | CTCTGCCTTTGAGAGGTTCCTG | AGCACTCGAAGCTGACGCCAAA |
| ***OGT*** | CAGGAAGGCTATTGCTGAGAGG | CGGAACTCACATATCCTACACGC |
| ***GDAP1*** | TGGACGCATTCCTTCAGCTCTC | CCAAGGCTCATTGTGCTCACTC |
| ***MCM2*** | TGCCAGCATTGCTCCTTCCATC | AAACTGCGACTTCGCTGTGCCA |
| ***CDC45*** | TGGATGCTGTCCAAGGACCTGA | CAGGACACCAACATCAGTCACG |
| ***CLK1*** | CACACGATAGTAAGGAGCATTTAG | GGCAGAACTGTGTTCATCCCAG |
| ***NUCKS1*** | GACGATAGTGACTATGGCAGTTC | CCTTTCACTGGACTTGGCGTCA |
| ***AURKA*** | GCAACCAGTGTACCTCATCCTG | AAGTCTTCCAAAGCCCACTGCC |
| ***LC3B*** | GAGAAGCAGCTTCCTGTTCTGG | GTGTCCGTTCACCAACAGGAAG |

**Table S2.** The primers for ChIP-PCR analysis.

|  | **Forward** | **Reverse** |
| --- | --- | --- |
| ***TRIAP1*** | CTCAGGAGTCCAGACAAGCCT | TCACTCTATCACCCAGGTTAGAGTG |
| ***LC3B*** | GCTCAGTGCAACCTCCGC | GCACTTAGGGAGGCCGAG |
