# Supplementary material for: The role of USP7-YY1 interaction in promoting colorectal cancer growth and metastasis
Source: Cell Death Dis. 2024 May 20;15(5):347. doi: 10.1038/s41419-024-06740-4 (PMC11106261; doi:10.1038/s41419-024-06740-4)

Figure 1C

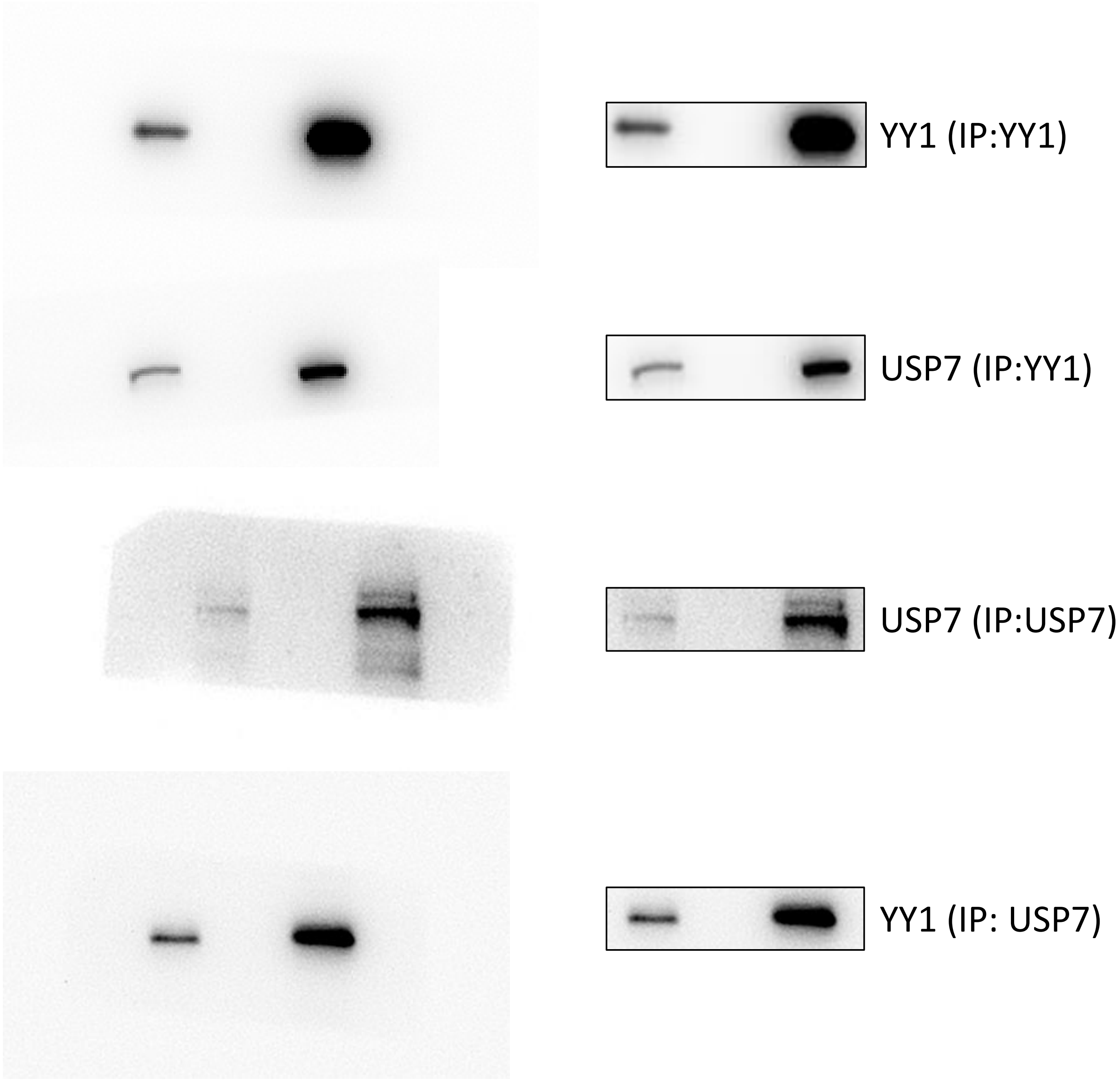

Figure 1D

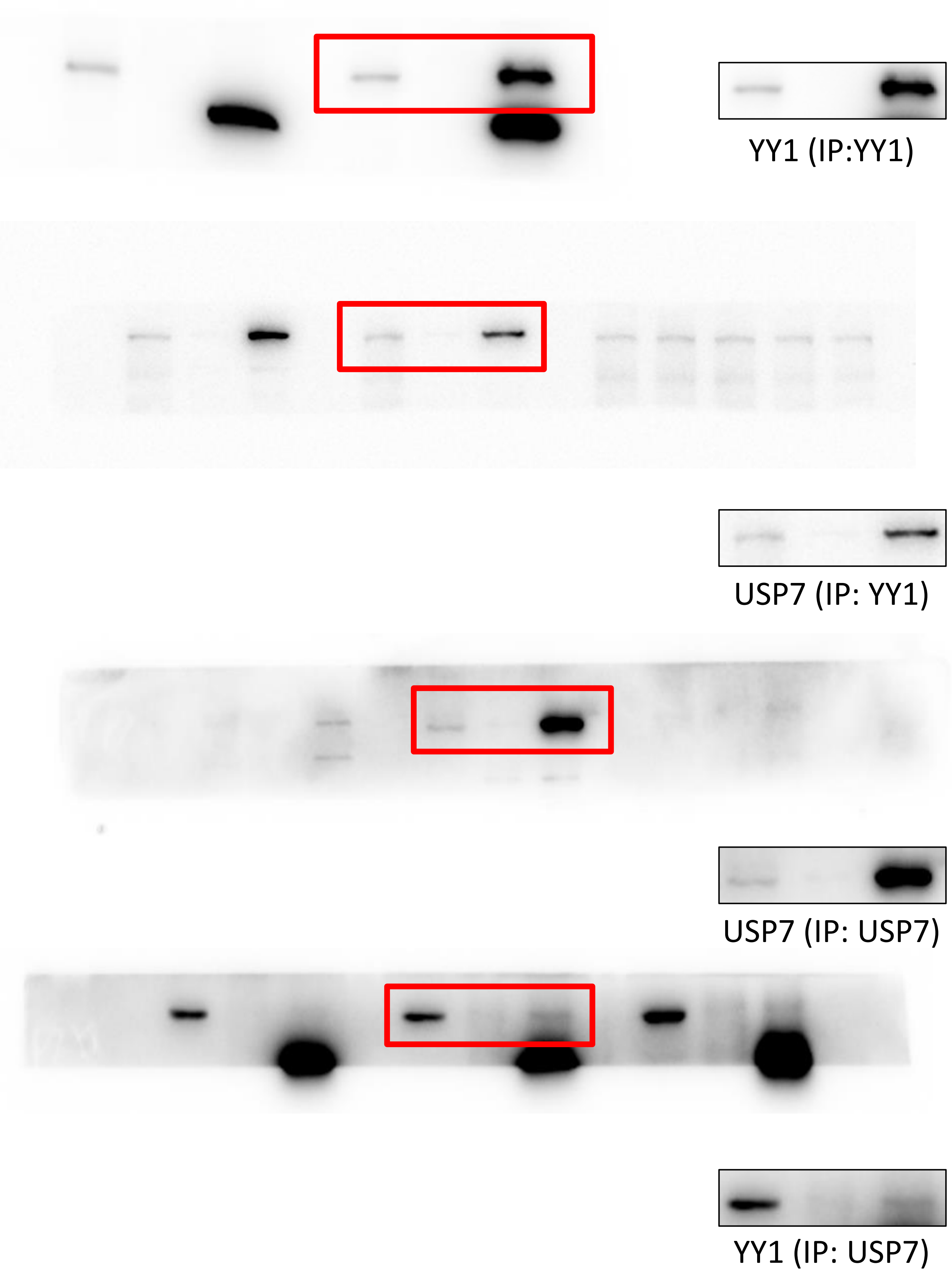

Figure 1E

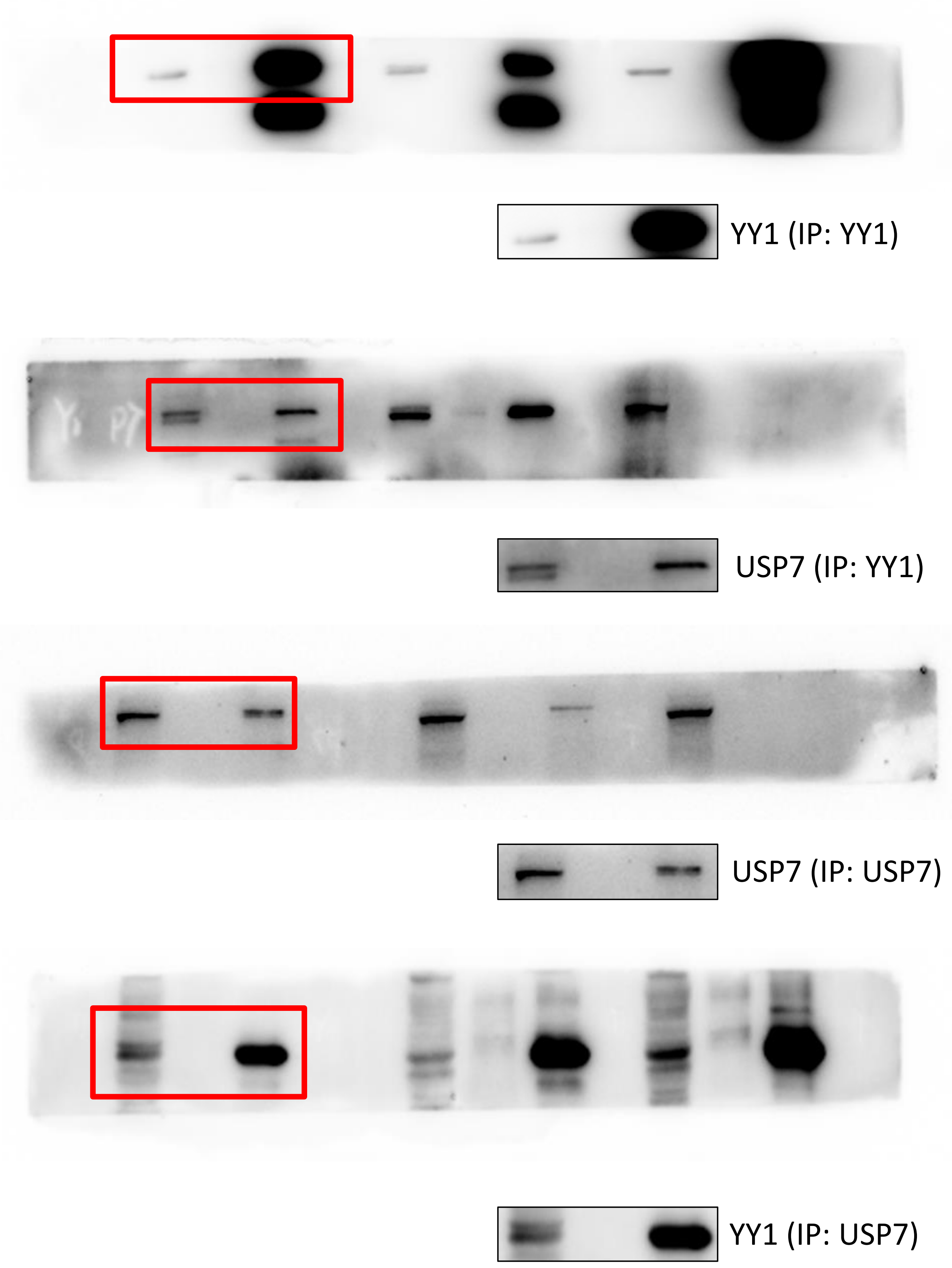

Figure 1F

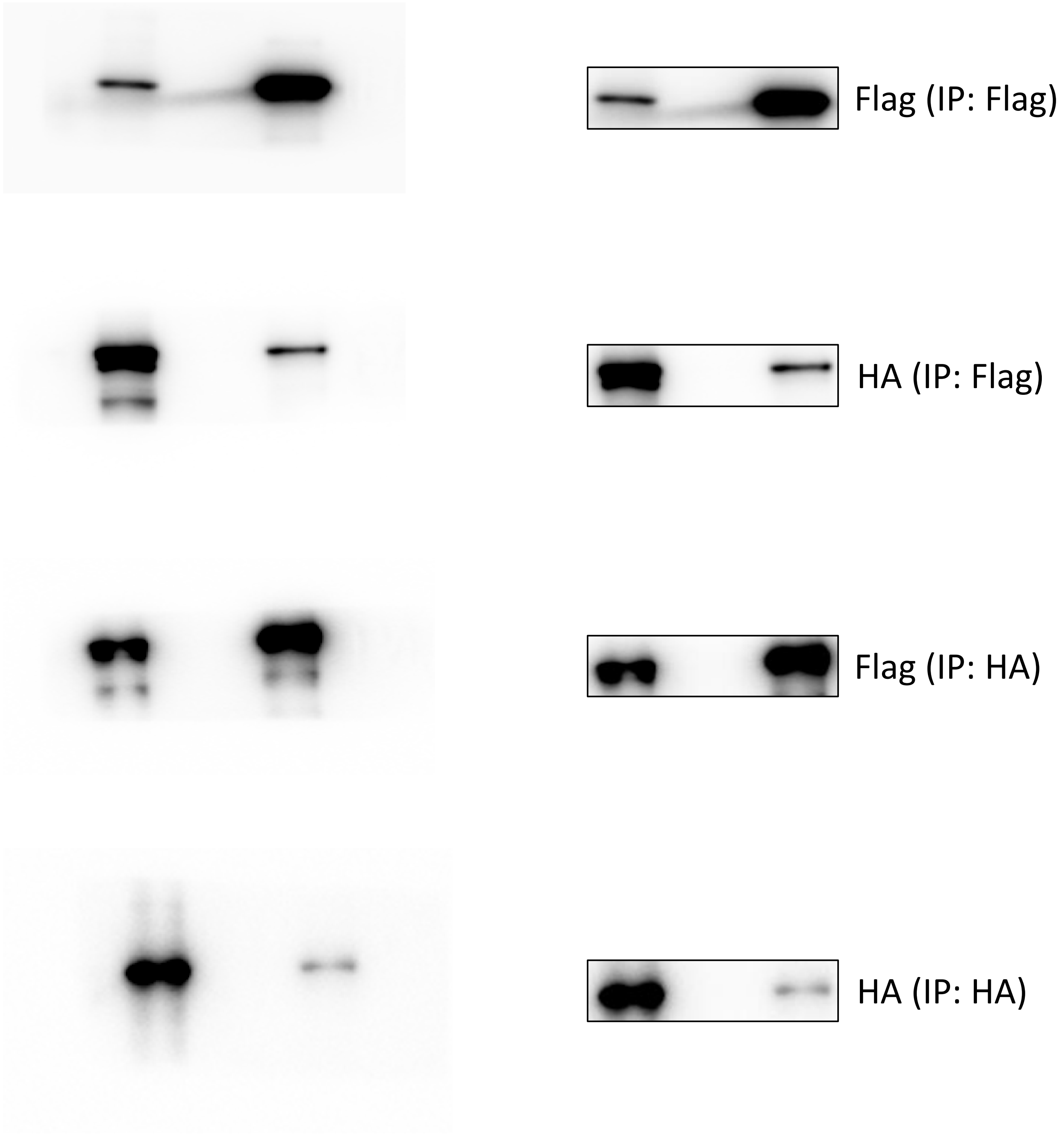

Figure 2A

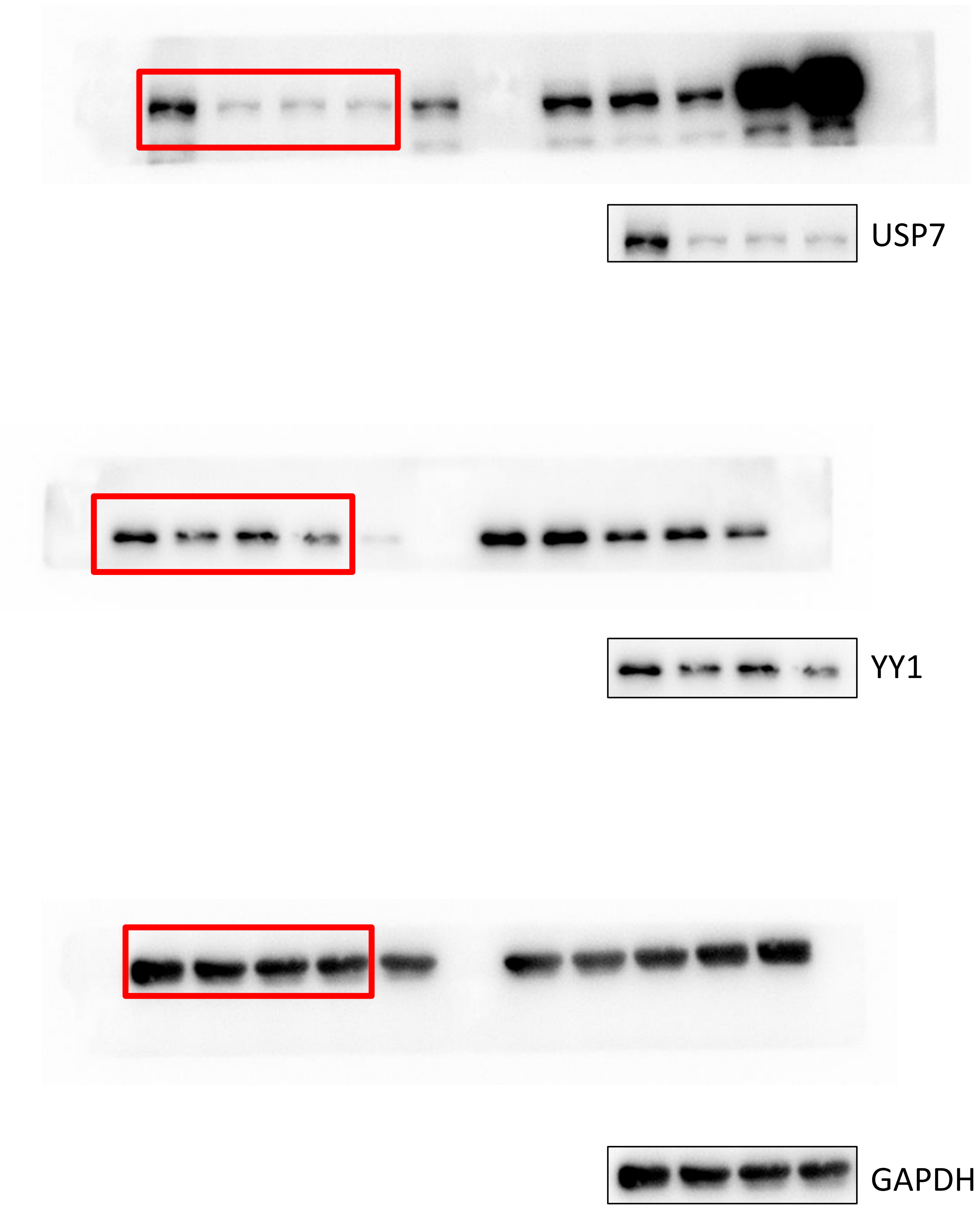

Figure 2B

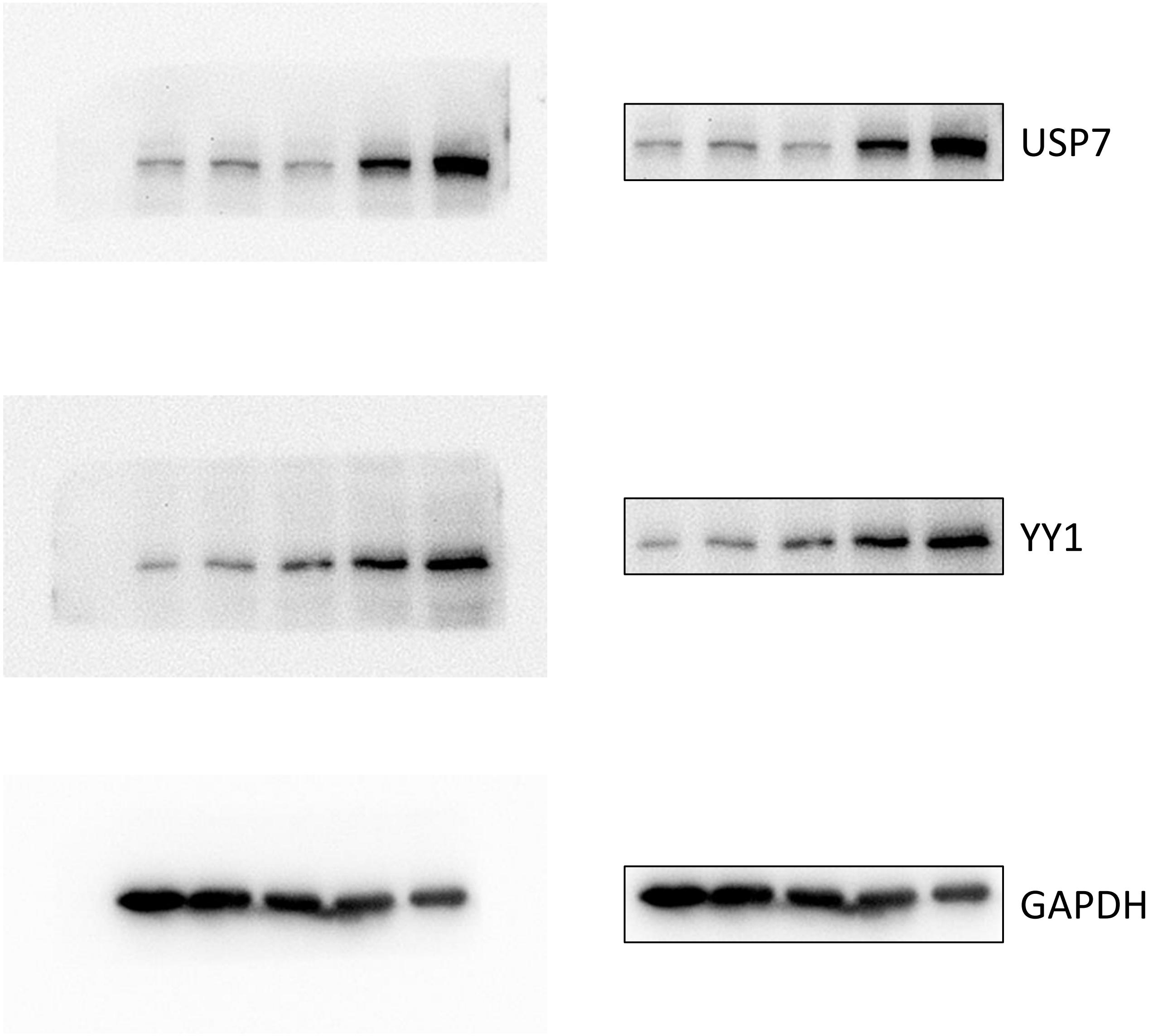

Figure 2D

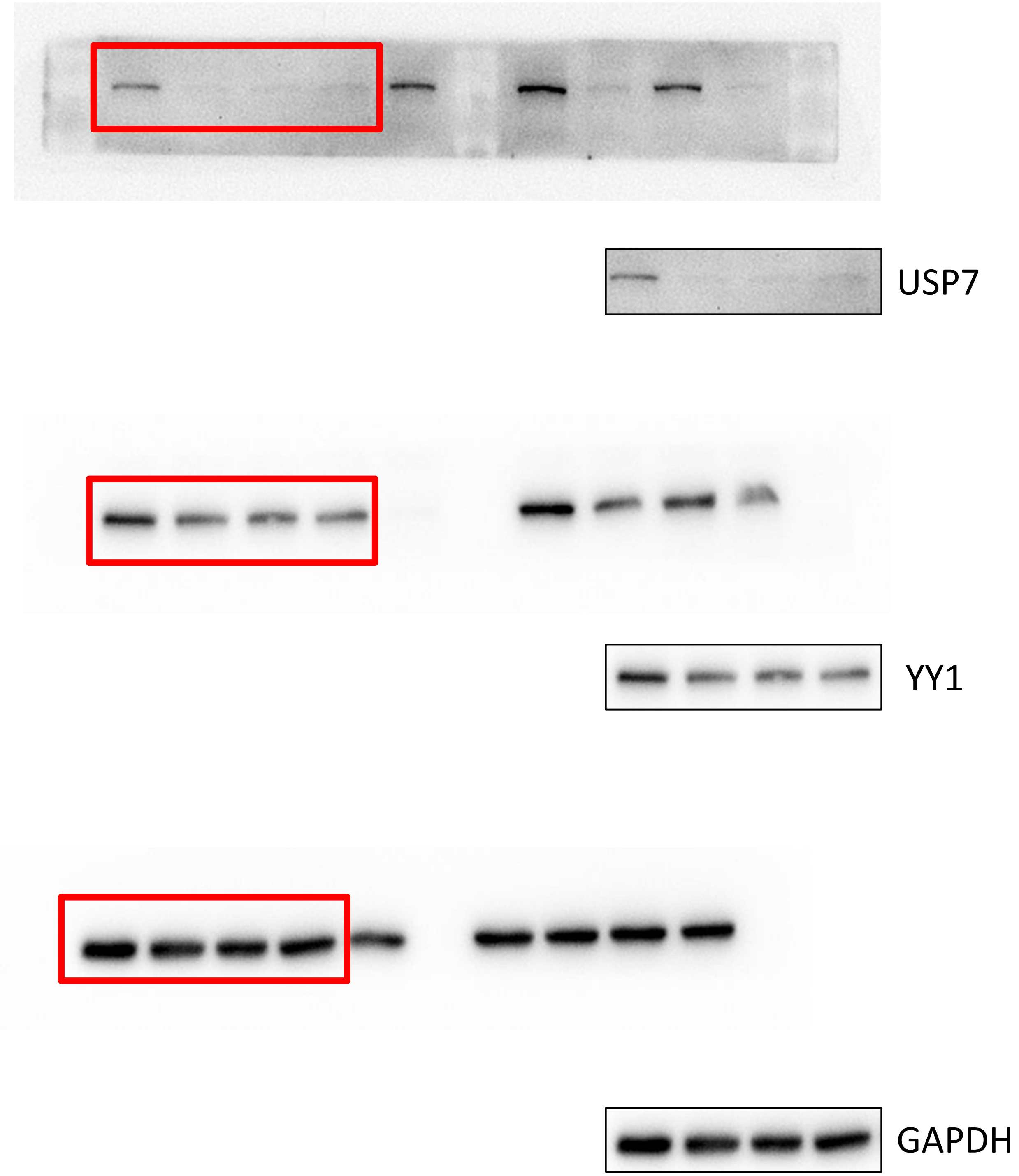

Figure 2E

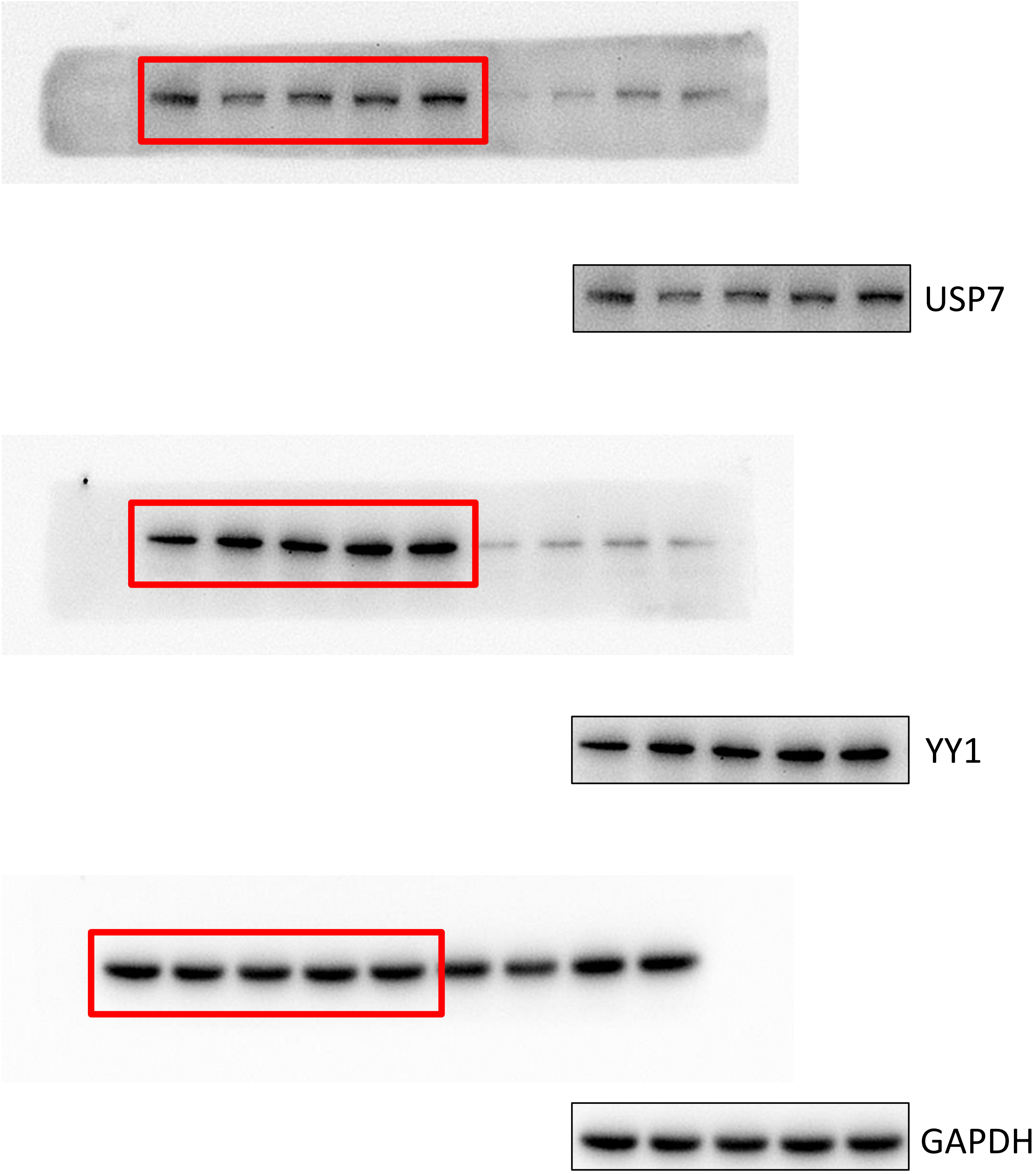

Figure 2G

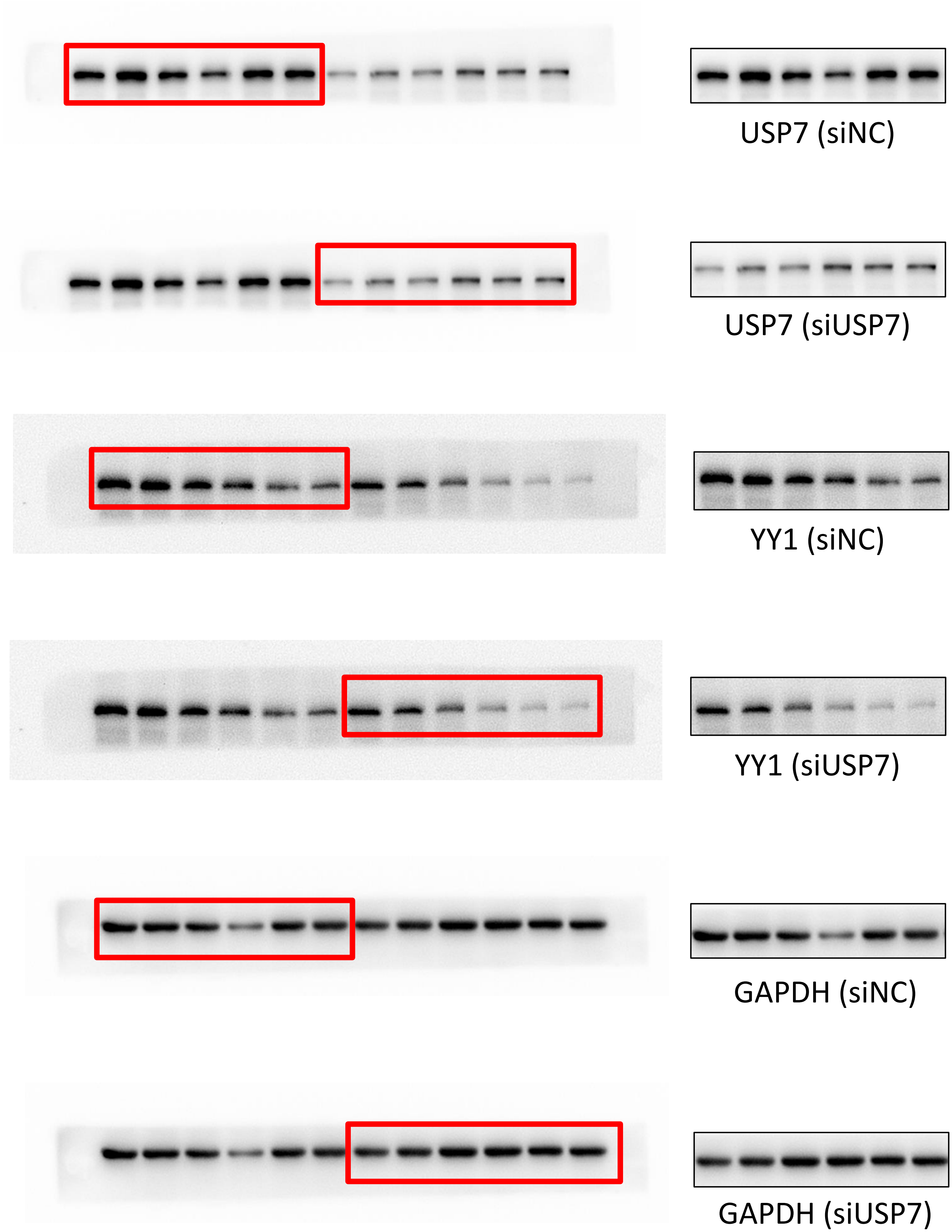
USP7 (siNC)
USP7 (siUSP7)
YY1 (siNC)
YY1 (siUSP7)
GAPDH (siNC)
GAPDH (siUSP7)

Figure 2H

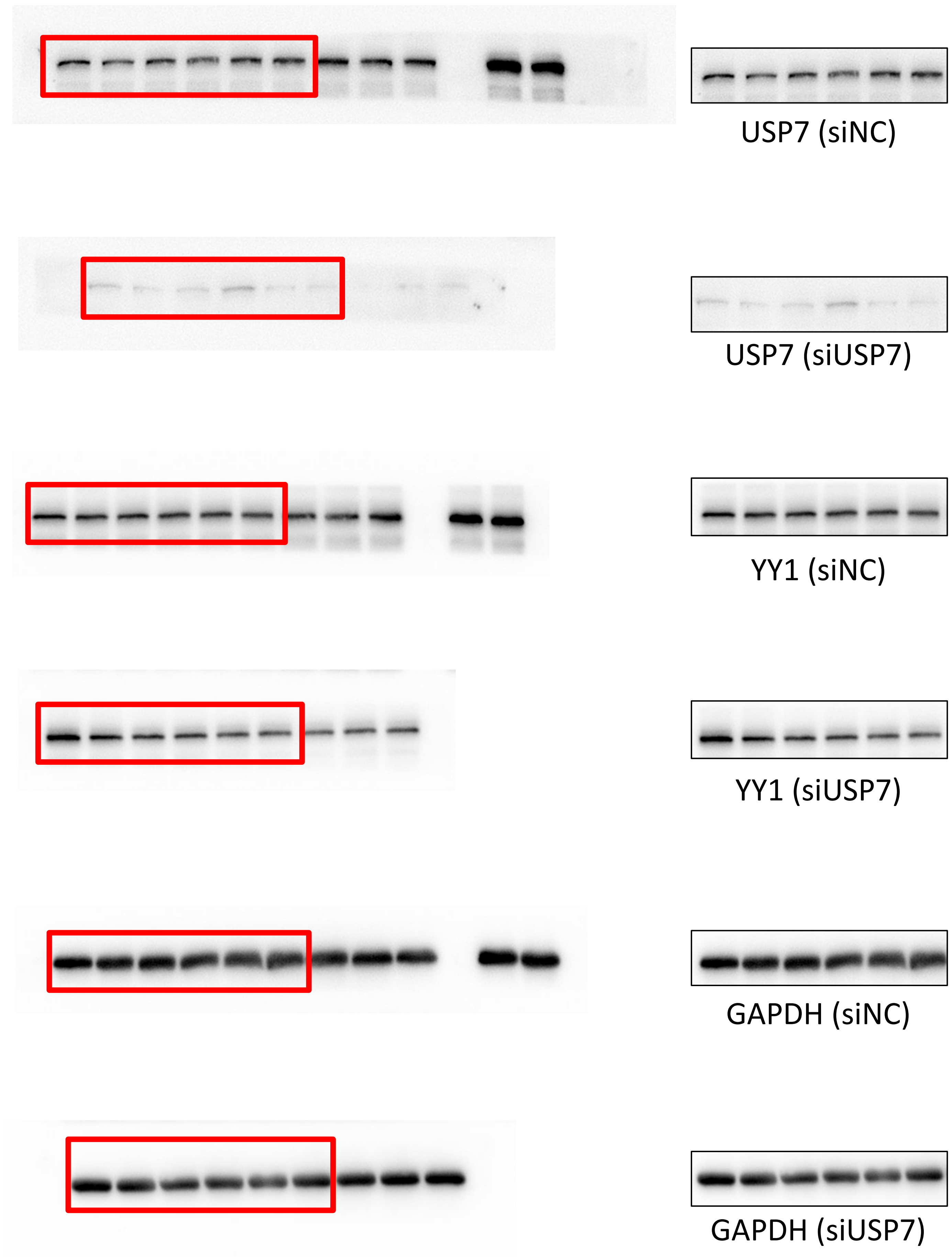

USP7 (siNC)
USP7 (siUSP7)
YY1 (siNC)
YY1 (siUSP7)
GAPDH (siNC)
GAPDH (siUSP7)

Figure 2I

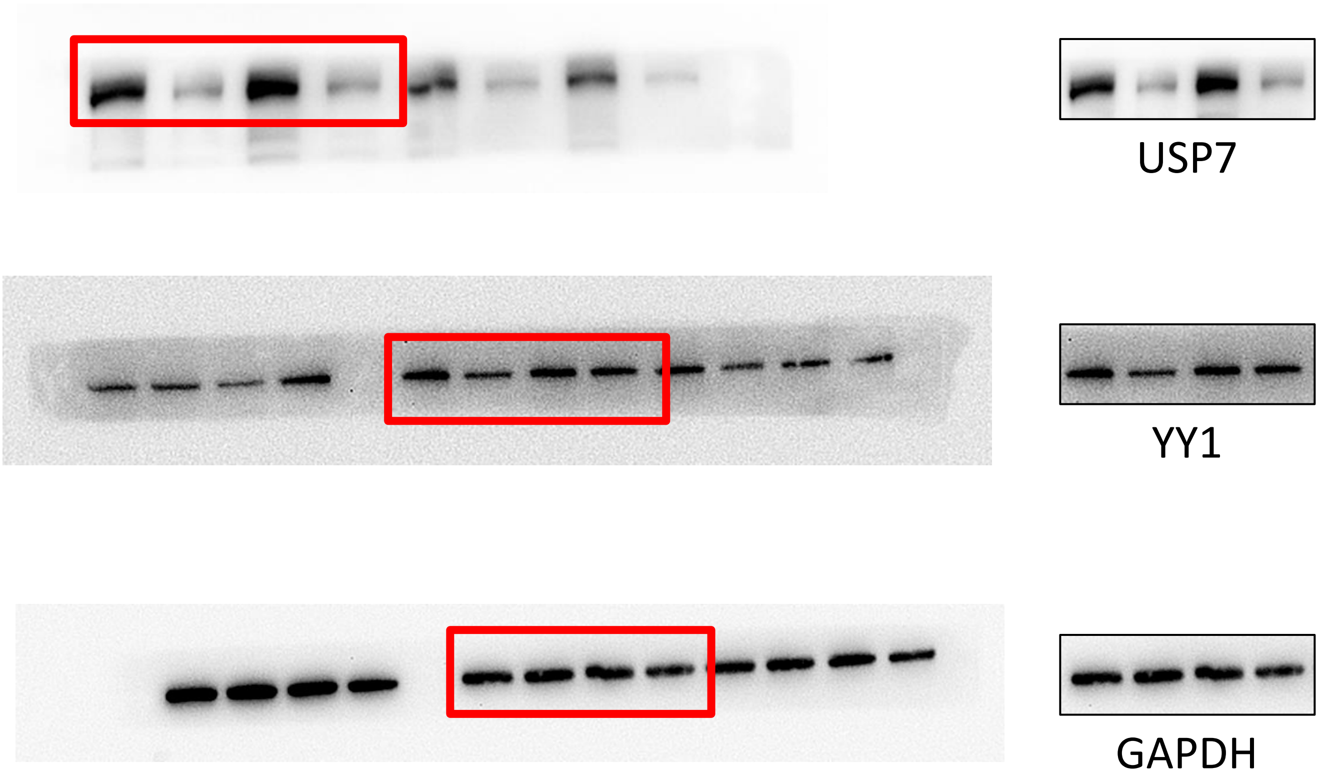

Figure 2J

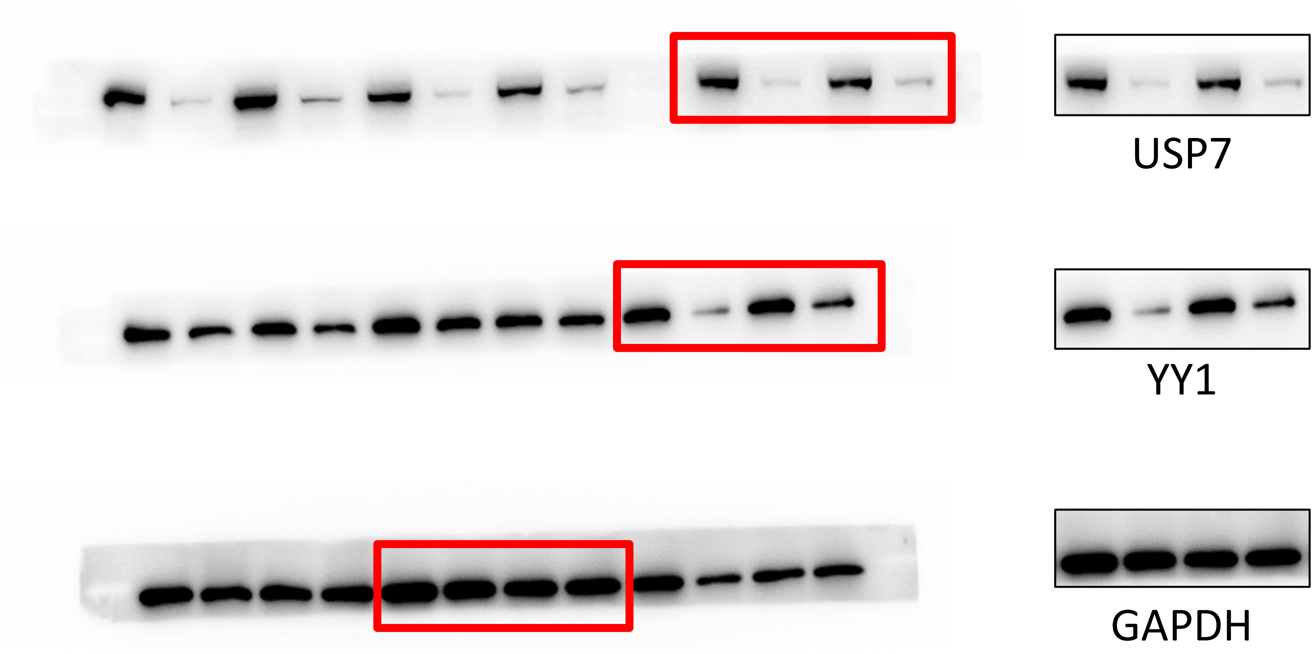

Figure 3A

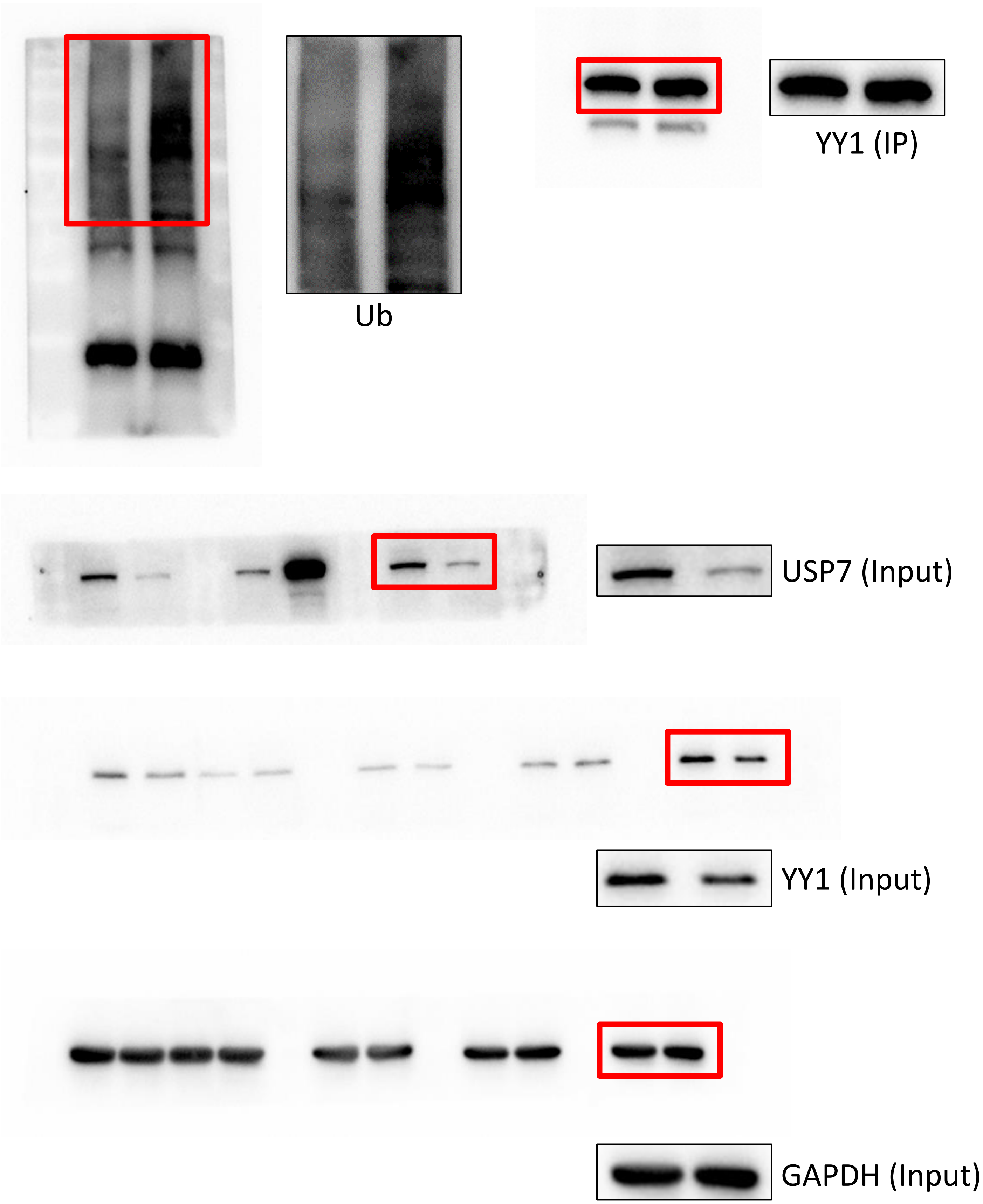

Figure 3B

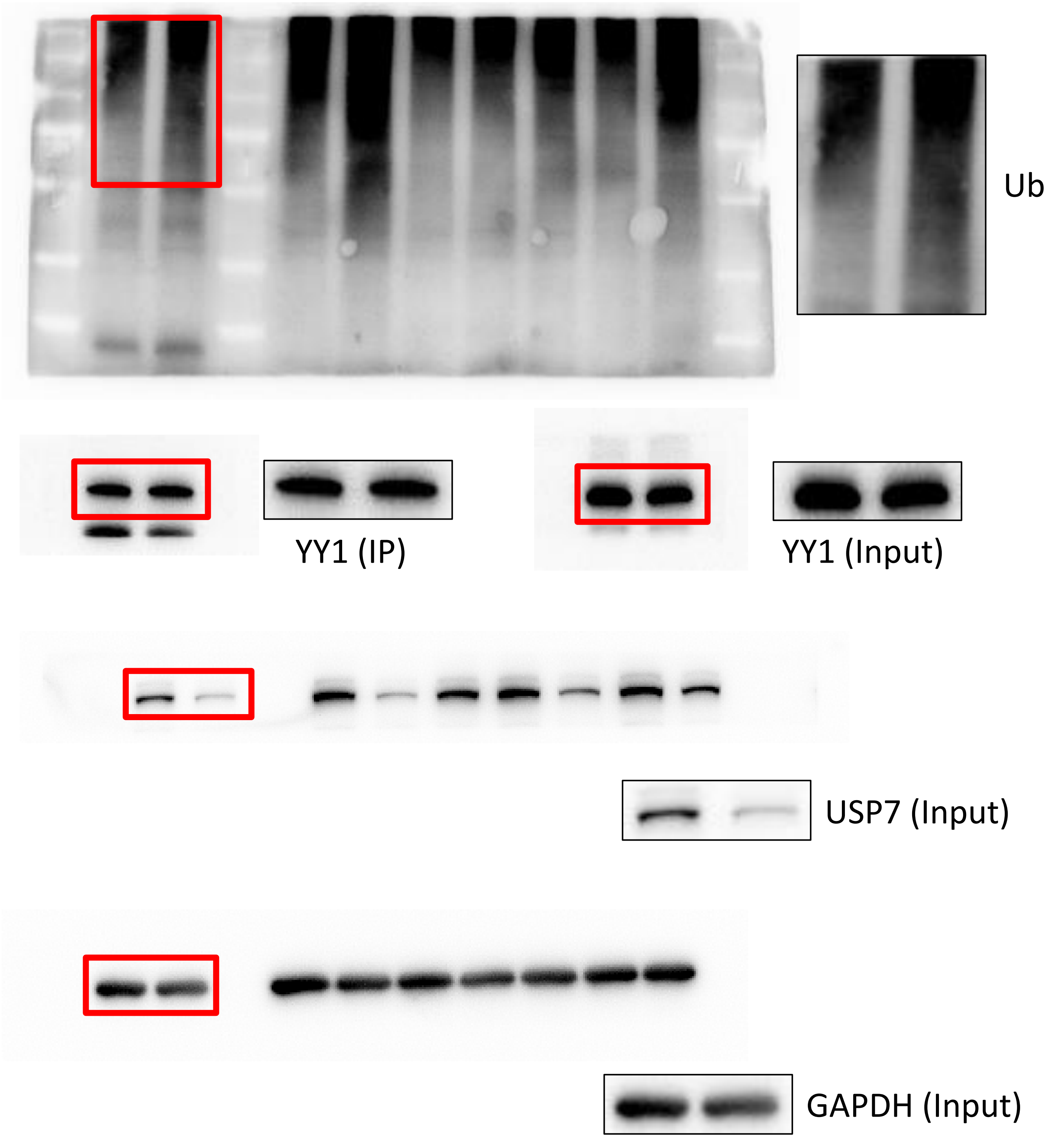

Figure 3C

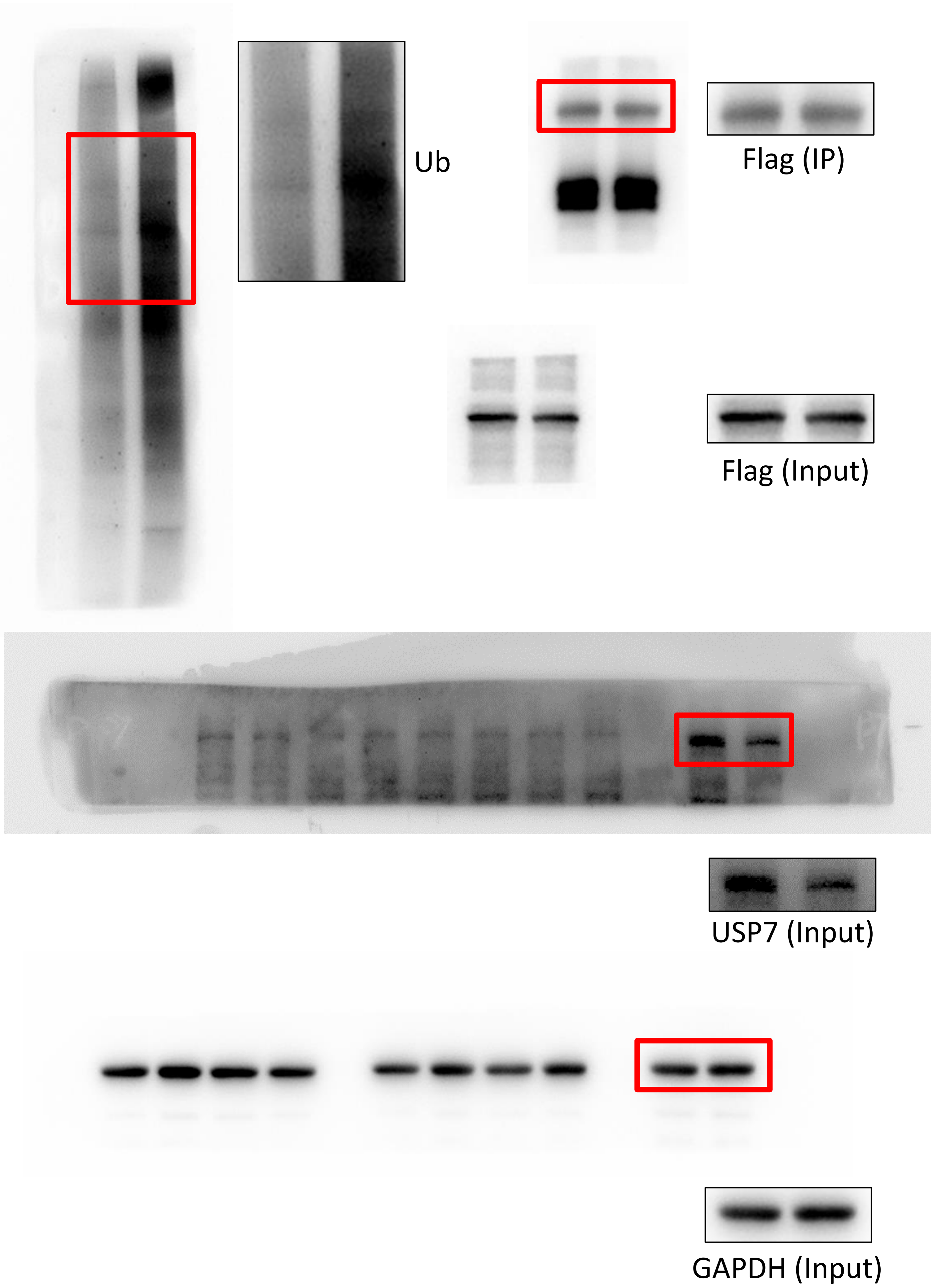

Figure 3D

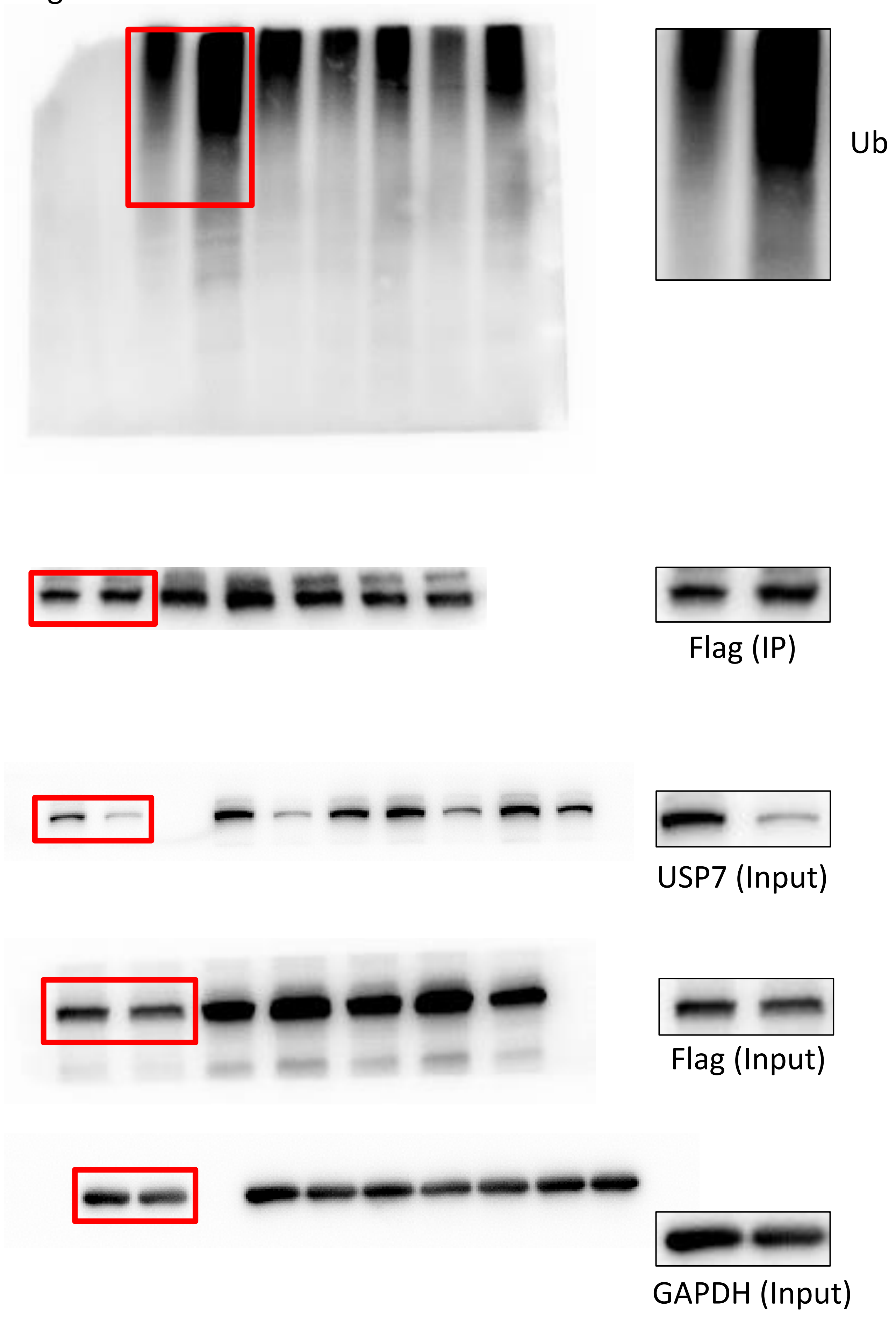

Figure 3E

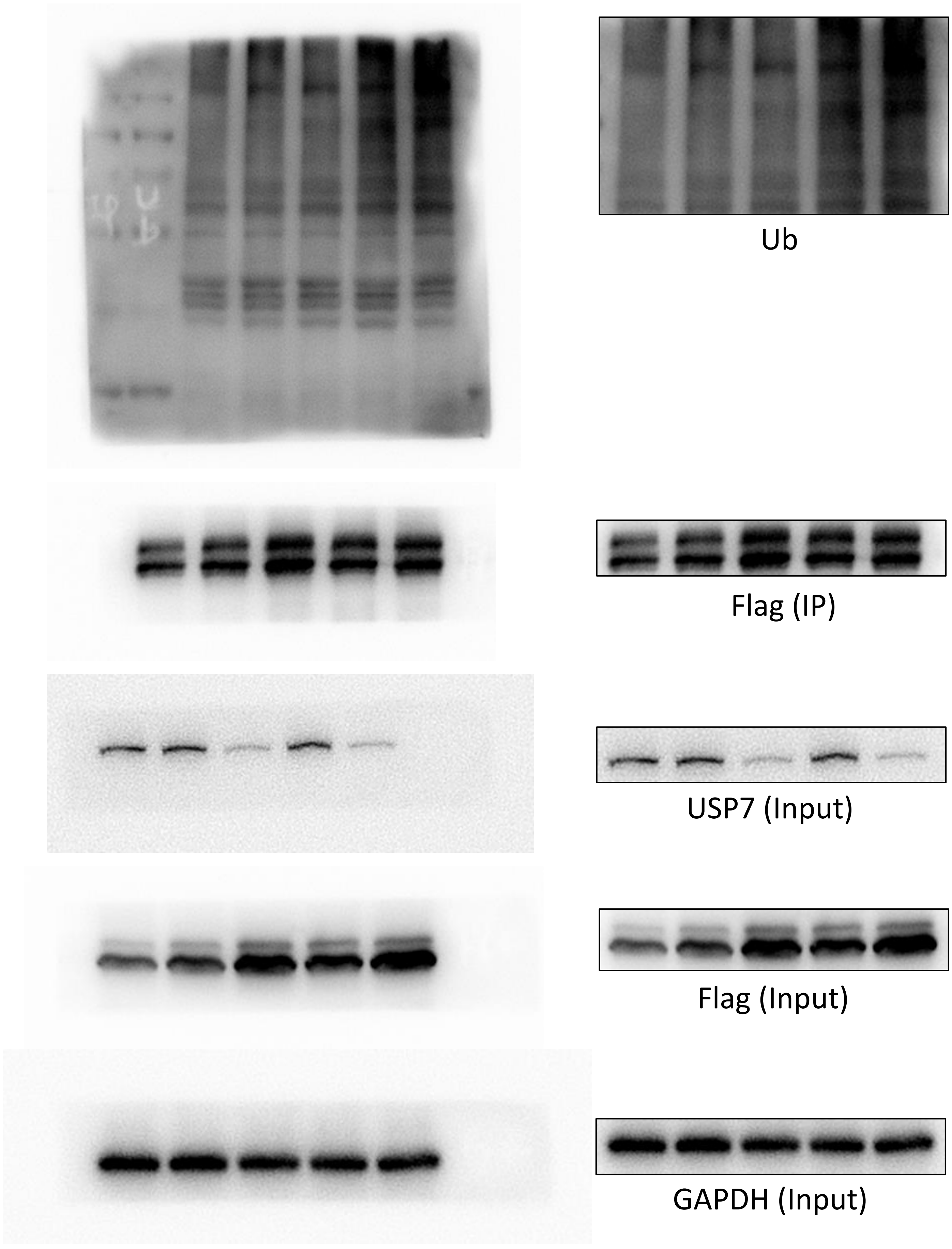

Figure 3F

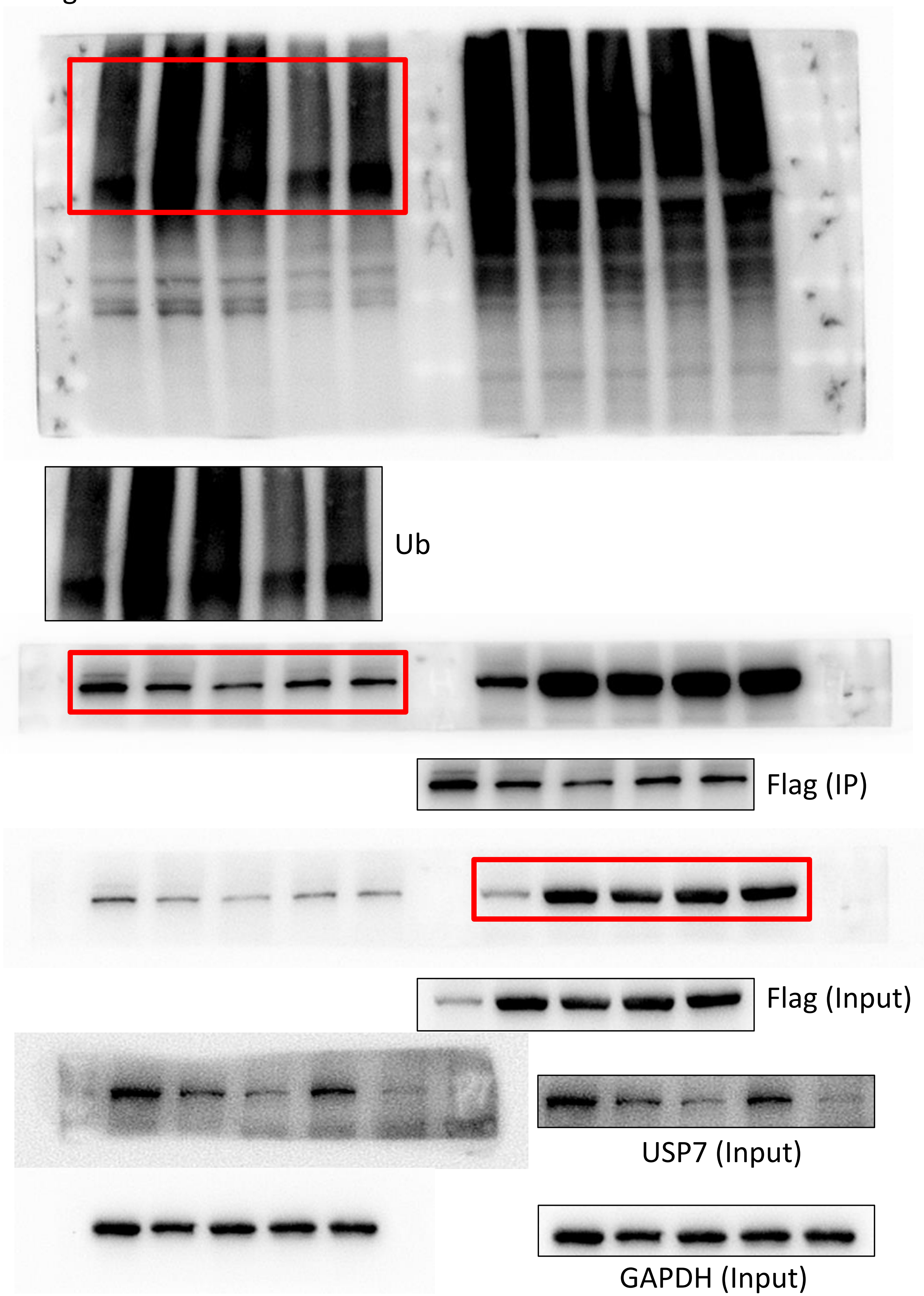

Figure 3G

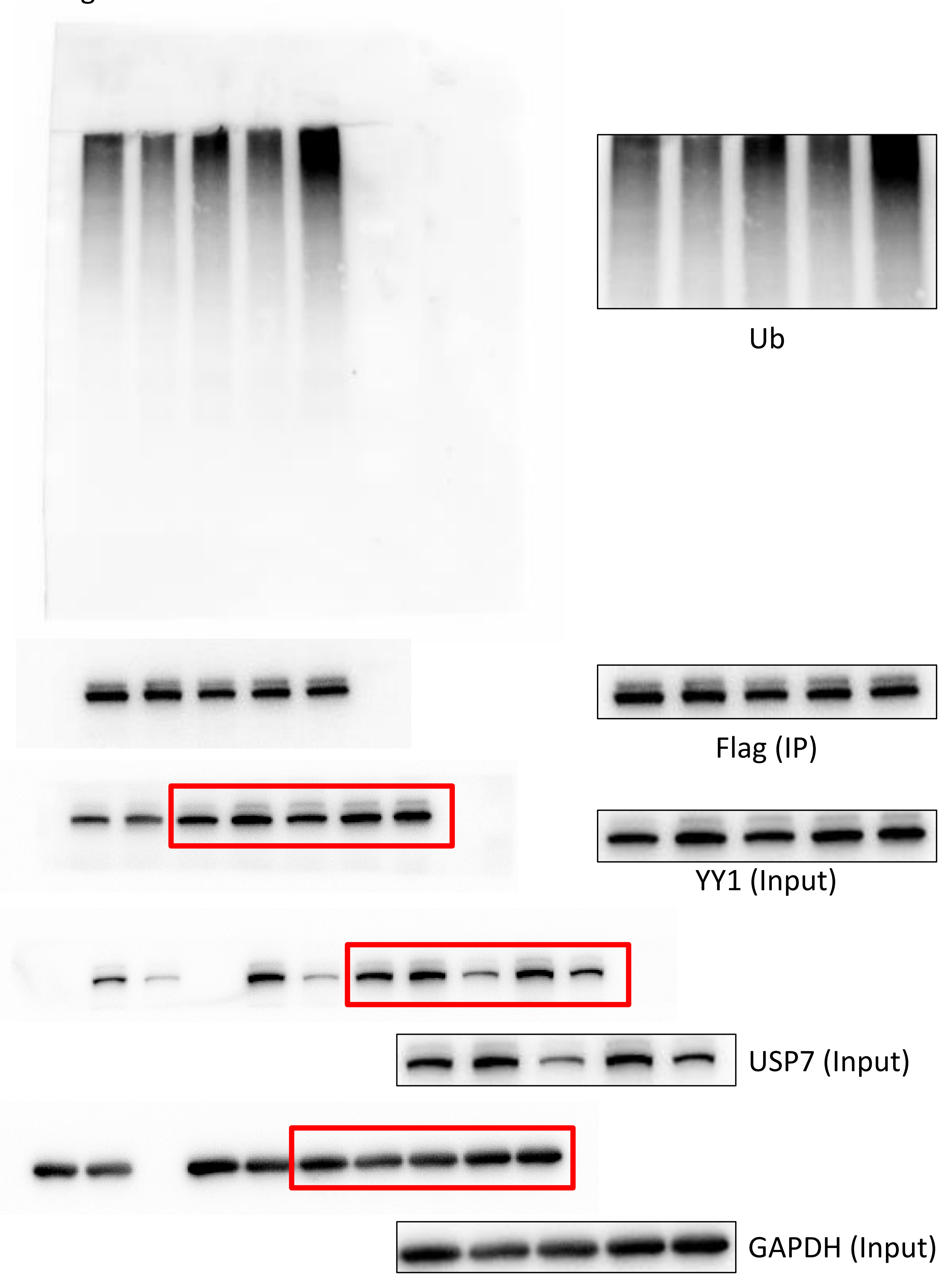

Figure 3H

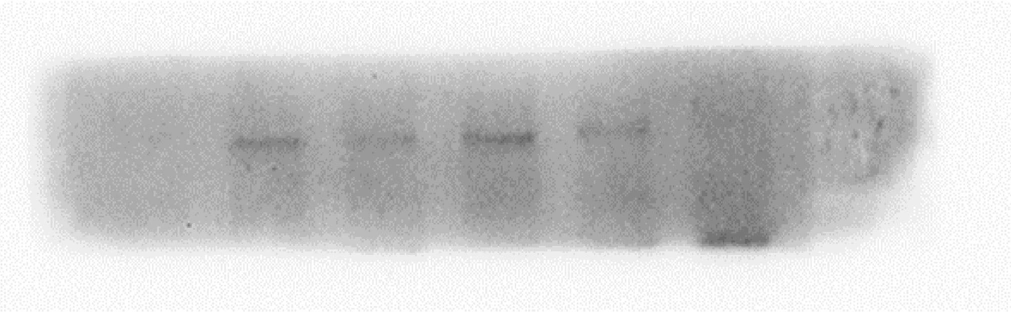

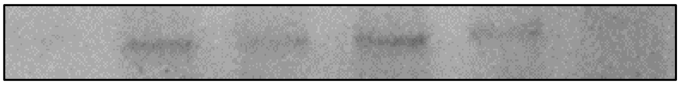

USP7

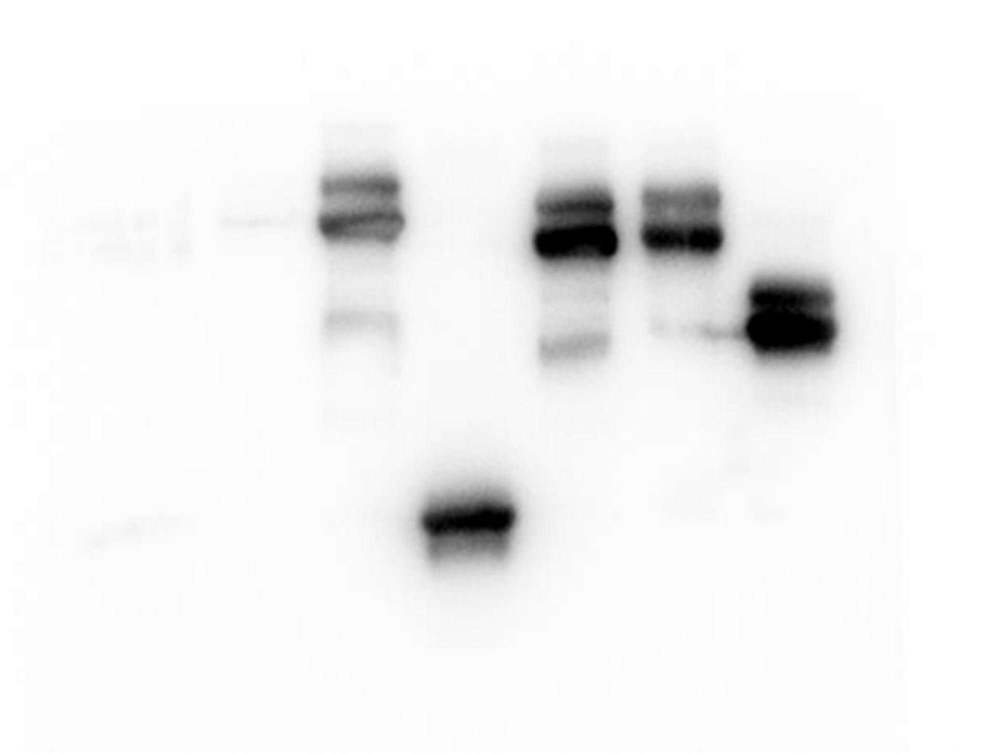

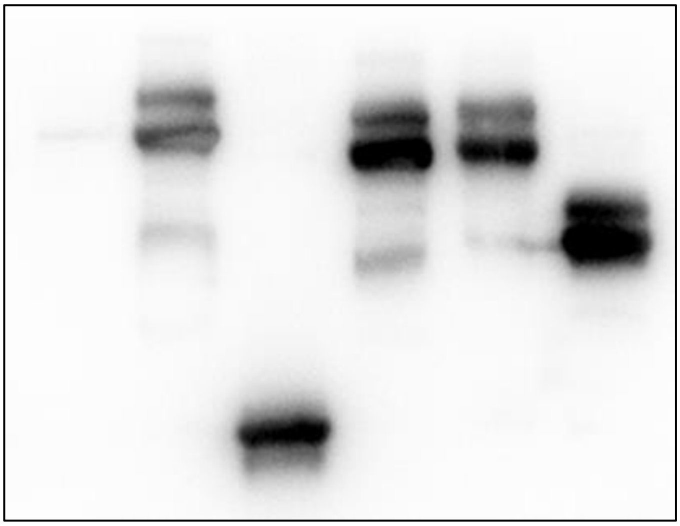

Flag (YY1)

Figure 4A

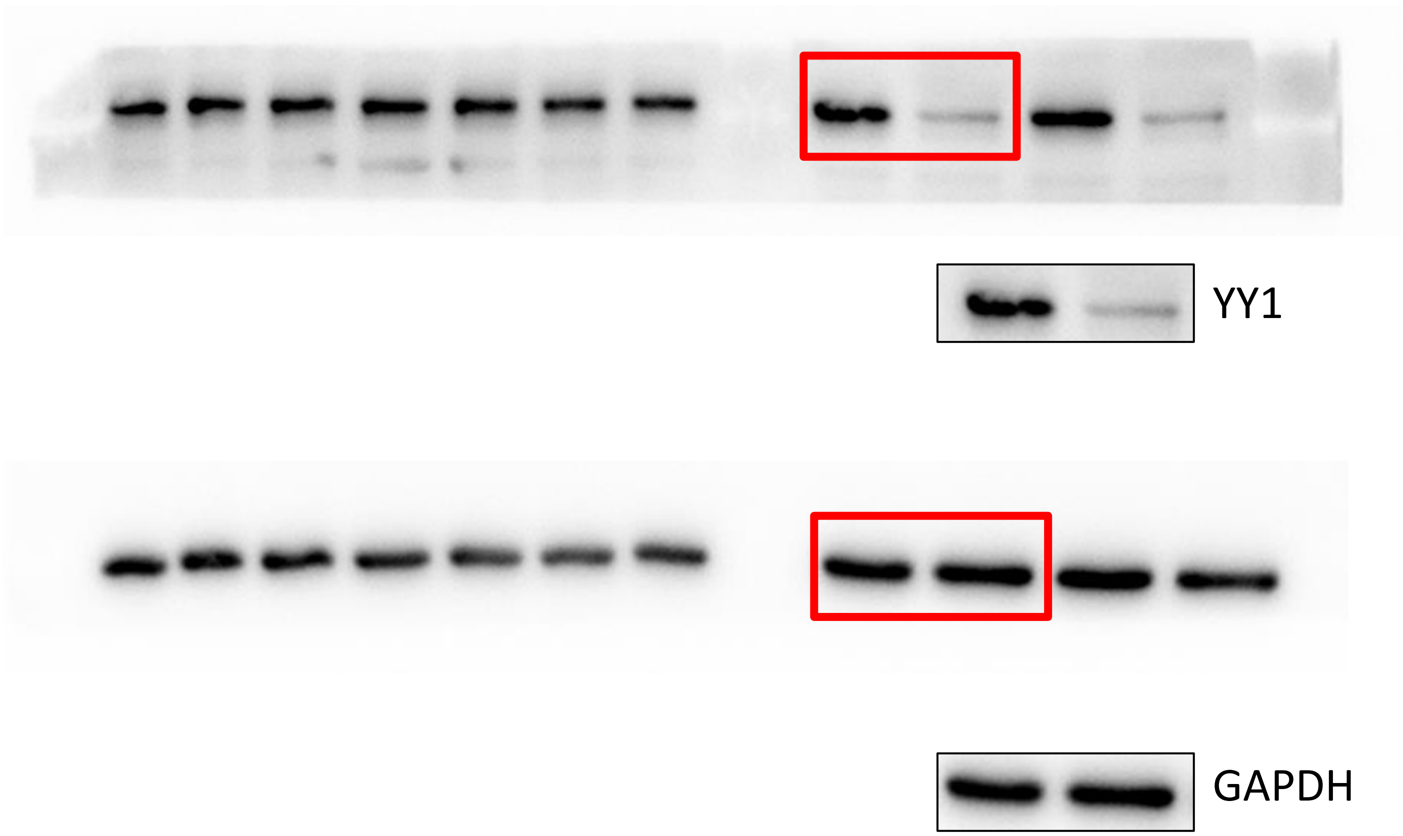

Figure 4H HCT116

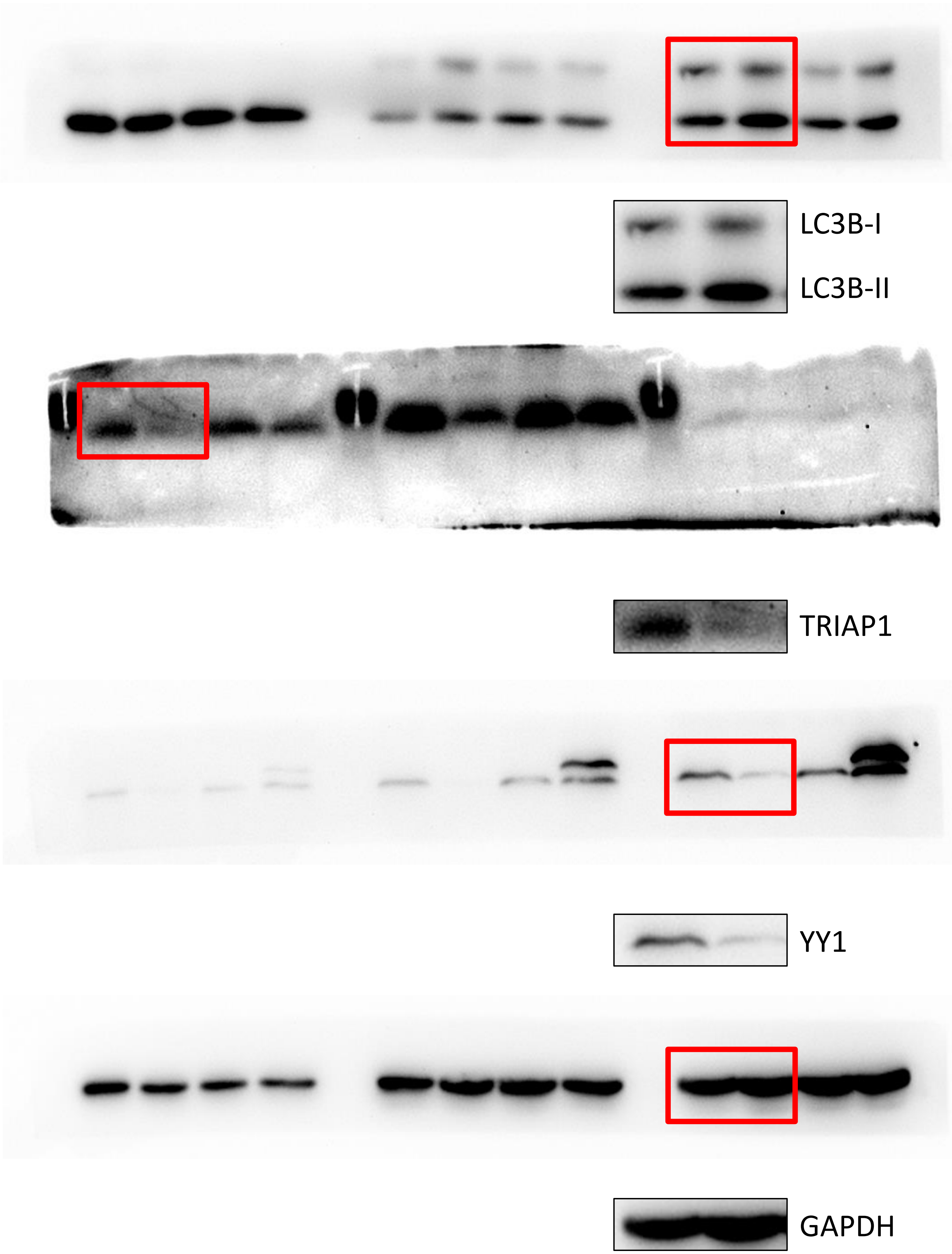

Figure 4H LOVO

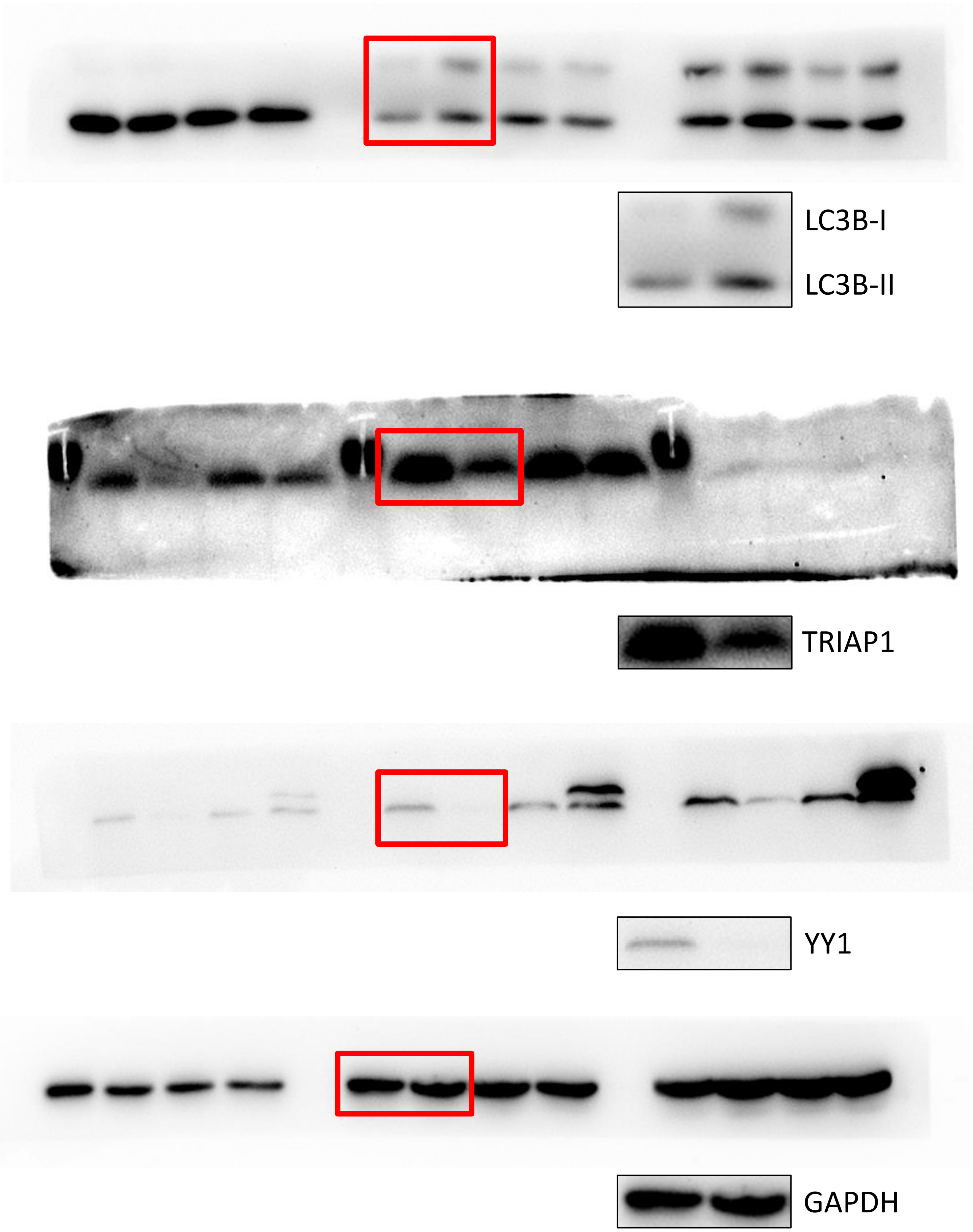

Supplementary Figure 1A

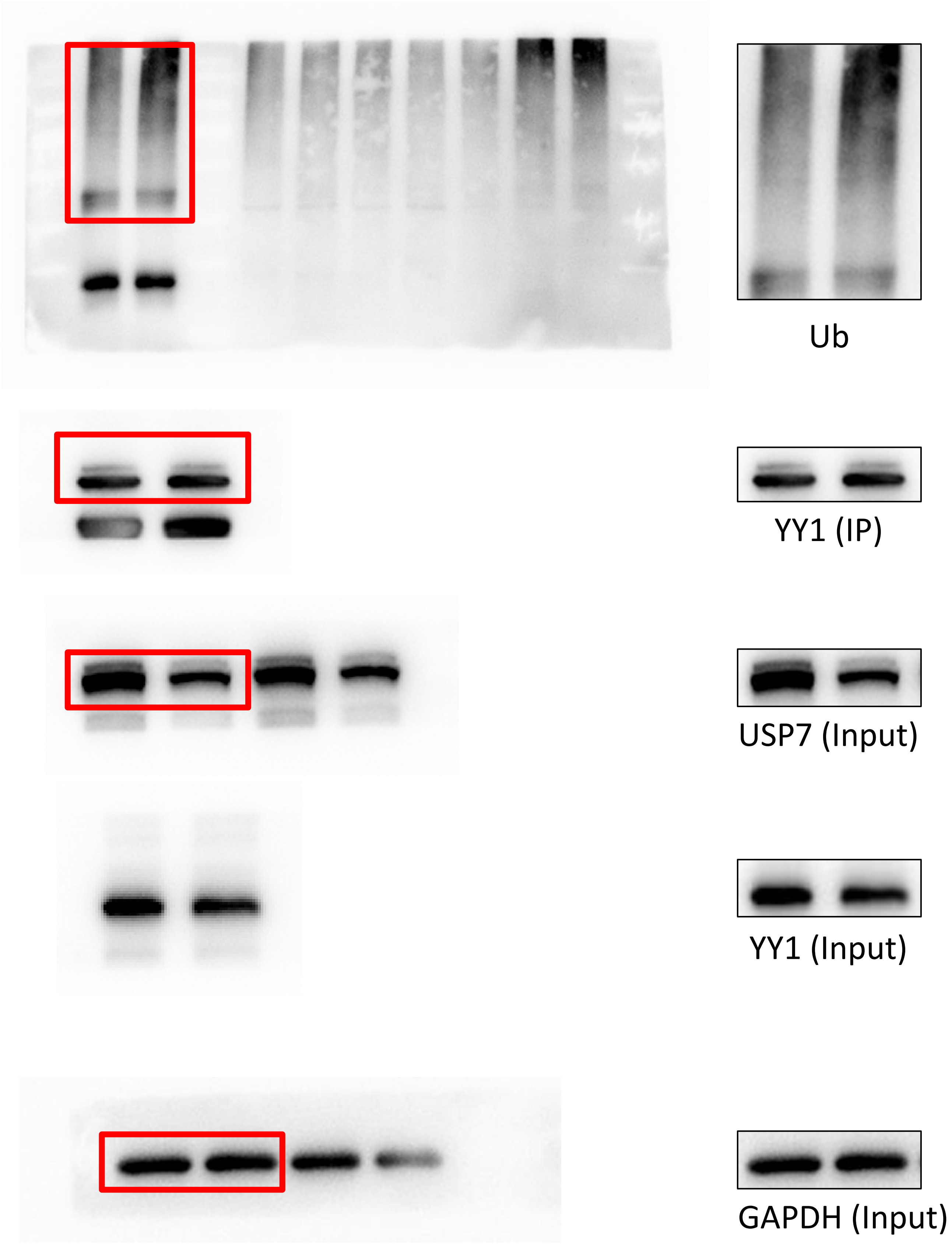

Supplementary Figure 1B

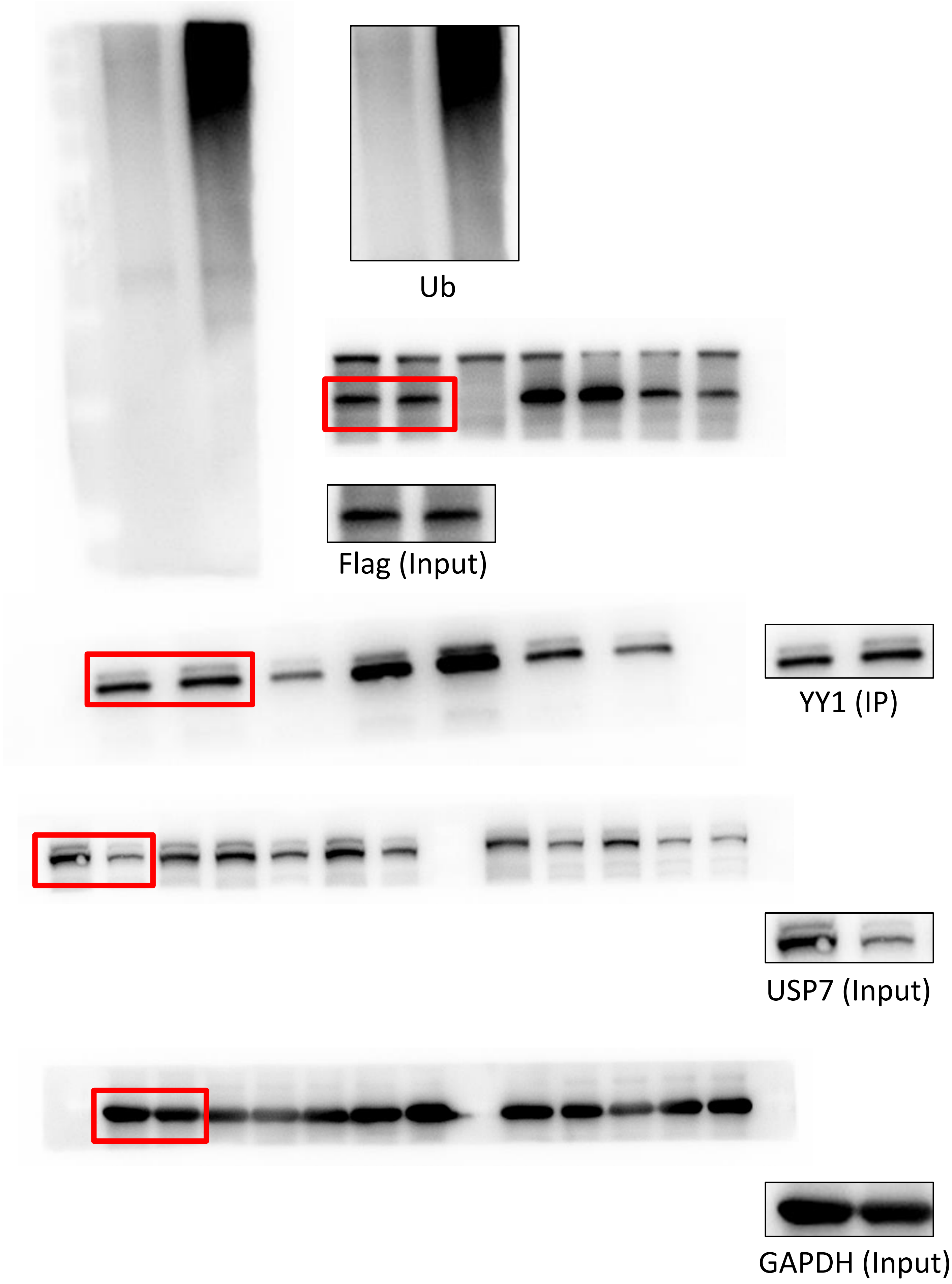

Supplementary Figure 1C

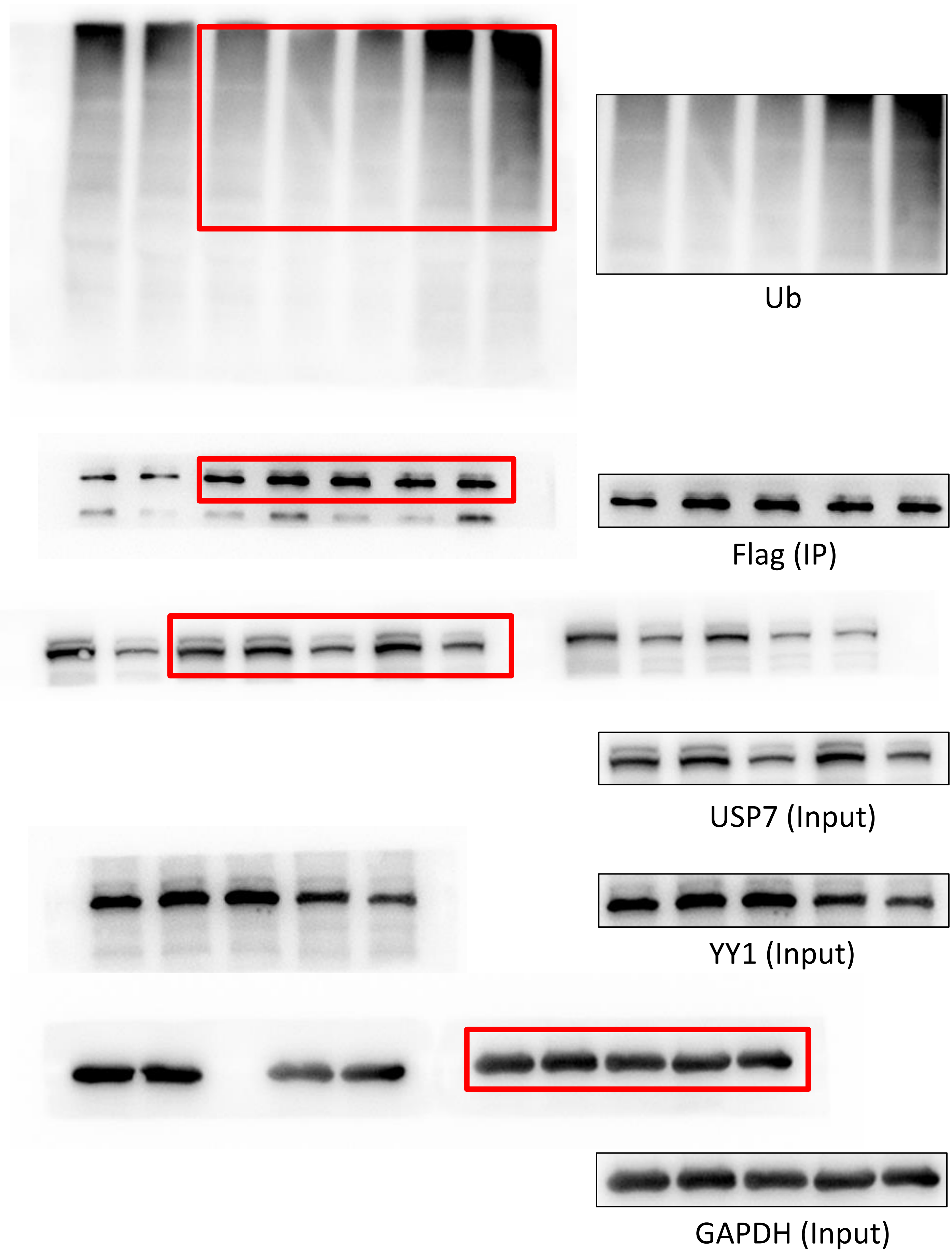

Supplement: Supplementary file 3 — Raw data of Western blotting [file 41419_2024_6740_MOESM3_ESM.pdf]
